# Heteroepitaxial chemistry of zinc chalcogenides on InP nanocrystals for defect-free interfaces with atomic uniformity

**DOI:** 10.1038/s41467-022-35731-2

**Published:** 2023-01-03

**Authors:** Yeongho Choi, Donghyo Hahm, Wan Ki Bae, Jaehoon Lim

**Affiliations:** 1 Department of Energy Science, Centre for Artificial Atoms, Sungkyunkwan University (SKKU), Suwon, 16419 Republic of Korea; 2 SKKU Institute of Energy Science and Technology (SIEST), Sungkyunkwan University, Suwon, 16419 Republic of Korea; 3 SKKU Advanced Institute of Nanotechnology (SAINT), Sungkyunkwan University, Suwon, Gyeonggi-do, 16419 Republic of Korea; 4 Chemistry Division, Los Alamos National Laboratory, Los Alamos, NM 87545 USA

**Keywords:** Electronic materials, Quantum dots, Synthesis and processing

## Abstract

Heteroepitaxy on colloidal semiconductor nanocrystals is an essential strategy for manipulating their optoelectronic functionalities. However, their practical synthesis typically leads to scattered and unexpected outcomes due to the intervention of multiple reaction pathways associated with complicated side products of reactants. Here, the heteroepitaxy mechanism of zinc chalcogenide initiated on indium phosphide (InP) colloidal nanocrystals is elucidated using the precursors, zinc carboxylate and trialkylphosphine selenide. The high magnetic receptivity of ^77^Se and the characteristic longitudinal optical phonon mode of ZnSe allowed for monitoring the sequence of epilayer formation at the molecular level. The investigation revealed the sterically hindered acyloxytrialkylphosphonium and diacyloxytrialkylphosphorane to be main intermediates in the surface reaction, which retards the metal ion adsorption by a large steric hindrance. The transformation of adsorbates to the crystalline epilayer was disturbed by surface oxides. Raman scattering disclosed the pathway of secondary surface oxidation triggered by carboxylate ligands migrated from zinc carboxylate. The surface-initiated heteroepitaxy protocol is proposed to fabricate core/shell heterostructured nanocrystals with atomic-scale uniformity of epilayers. Despite the large lattice mismatch of ZnS to InP, we realised a uniform and interface defect-free ZnS epilayer (~0.3 nm thickness) on InP nanocrystals, as evidenced by a high photoluminescence quantum yield of 97.3%.

## Abstract (web summary)

Heteroepitaxy on colloidal nanocrystals often yields defective heterostructures due to intricate reaction pathways. Here, the authors decode the surface chemistry at the molecular level to realise defect-free interfaces with atomic uniformity.

## Introduction

Heteroepitaxy on colloidal semiconductor nanocrystals (NCs) is an essential strategy for manipulating their optoelectronic functionalities, such as band gap^1^, emission bandwidth^2,3^, and ultrafast carrier dynamics^4–7^. For size-, shape-, and composition-controlled NCs, the adoption of heterointerfaces, with partial or entire epitaxy of different materials, allows for manipulating the distribution of carrier wavefunctions and their ultrafast dynamics. For example, a type-I core/shell heterostructure confines the carrier wave functions to the NC interior, thus improving the photoluminescence quantum yield (PL QY)^1,8–11^. Type-II heterojunctions can adjust the oscillator strength and transition energy of excitons by controlling their spatial distribution in NCs^12–14^. The smoothed confinement potential at the interface is capable of accessing multicarrier dynamics occurring in the 10–100s ps range^7,15–17^.

The heteroepitaxy mechanism on colloidal NCs is described based on the modified La Mer plot; the formation of an epitaxial layer results from the heterogeneous nucleation and growth of monomers (i.e. the smallest units made up of cations, anions, and/or ligands) on surface^18^. For instance, in the case of the monomer generation between metal carboxylates and trialkylphosphine chalcogenides, the nucleophilic attack of metal anions followed by the oxidation of trialkylphosphine is thought to be the major reaction pathway^19,20^. However, the reduction of metal carboxylate^21^ and dissociation of trialkylphosphine chalcogenide^22^ are also likely to occur at elevated temperatures, which also participates in monomer formation. Additives such as surface ligands^23^ or reducing agents^24^ modulate the reactivity of monomers on the NCs’ facets. In the practical synthesis of heterostructured NCs, those numerous reaction pathways simultaneously involve to the heteroepitaxy. As a result, regardless of using identical reactants, heterostructured NCs exhibit scattered and unexpected outcomes in terms of morphological uniformity, conformality, and interfacial defects. For example, significant challenges were encountered in the heteroepitaxy of ZnSe and/or ZnS on InP NCs to increase their PL QY. Many studies have already achieved PL QY exceeding 0.8 or almost unity for CdSe-based heterostructured NCs^25,26^, whereas that of InP-based heterostructured NCs typically yielded 0.4–0.7^9,10,27,28^ and near unity was laboriously reached by Jang and co-workers^29,30^.

The complexity of the heteroepitaxy process can be eliminated by channelling a singular pathway. Several elaborate schemes for colloidal NCs have been developed to separately control the metal ion adsorption and epilayer formation. Among these, successive ionic layer adsorption and reaction^4,31,32^ and colloidal atomic layer deposition^33,34^ have virtually accomplished this goal. However, except for several evident cases utilising the adsorption of ionic species in polar mediums^33,34^, there are numerous notes of interrogation regarding the surface chemistry of heteroepitaxy, such as metal ion migration from organometallic precursors to the surface, precursor conversion and/or decomposition on the surface, bond cleavage and linkage between adatoms and ligands, crystallisation of the epitaxial layer, and so on. Those question marks must be resolved to enable the rational design of a heteroepitaxy protocol for colloidal NCs, with the aim of developing an epilayer exhibiting coherency, conformality, and defect-free heterointerfaces.

In this study, we unveiled the heteroepitaxy mechanism of zinc chalcogenides on InP NCs at the molecular level. To exploit the high magnetic receptivity of ^77^Se and the characteristic phonon mode of ZnSe, our investigations were mainly centred on the reaction of zinc carboxylate and trialkylphosphine selenide on the InP NCs. Multilateral nuclear magnetic resonance (NMR) analysis clarified the reaction pathway of metal ion adsorption in which a carboxylate ligand was transferred to trialkylphosphine to generate acyloxytrialkylphosphonium and diacyloxytrialkylphosphorane intermediates. Raman scattering and X-ray diffraction (XRD) expatiated on the detailed aspects of ZnSe epilayer formation, for instance, the conversion of ZnSe adsorbates to a crystalline epitaxial layer, surface oxides as the main culprit obstructing the epilayer growth, and re-oxidation of the surface by zinc carboxylate. Based on a profound understanding of the heteroepitaxy mechanism, we designed the precision heteroepitaxy scheme for core/shell heterostructured NCs. Despite the most lattice-mismatched ZnS against InP, InP/ZnS NCs yielded a PL QY of 97.3% with a single ZnS epilayer (~0.3 nm) owing to the atomic scale uniformity and the absence of interfacial defects. This result signifies that an understanding of the surface chemistry of heteroepitaxy is essential to explore synthesis schemes for defect-free heterostructured NCs.

## Results

### Surface reaction of precursors and intermediate species

To investigate these challenges, we chose the widely used zinc oleate [Zn(OA)_2_] and trioctylphosphine selenide (SePR’_3_; R’ is octyl) as the heteroepitaxy precursors to analyse the sequence of ZnSe epilayer formation on the oleate-capped InP NCs (InP–OA; the radius of 2.4 or 3.3 nm). Notably, InP NCs have been extensively studied since the inception of semiconductor NCs and are of practical importance in the display industry as colour-converting materials^30,35^. Although the PL QY of InP NCs has recently approximated unity through InP/ZnSe/ZnS core/shell/shell heterostructures^29,30,36,37^, detailed research on the heteroepitaxy chemistry at the molecular level has not been established. ZnSe was selected from among the various zinc chalcogenides for the following reasons. First, ^77^Se features high natural abundance and magnetic receptivity, enabling the convenient tracing of its bond cleavage and linkage in SePR’_3_ using NMR. Second, the longitudinal optical phonon (LO) mode of ZnSe does not overlap with that of InO_*x*_ and InP, allowing the formation of the ZnSe epilayer to be observed via Raman scattering. To prevent side effects by water contamination (e.g. oxidation of metal carboxylates^38^, surface oxidation of InP NCs^39^, or alteration of growth kinetics^40^) or free oleic acid (e.g. water release by ketonisation^39^ or macromolecular complex formation^41^), all chemicals were carefully dried and stoichiometric Zn(OA)_2_ stock solution (i.e., Zn:oleic acid = 1:2 in molar ratio) was used. Free oleic acid was untraceable from ^1^H NMR of Zn(OA)_2_ stock solution, for example, the disappearance of acidic proton and increased diffusion coefficient of methine proton in Zn(OA)_2_ compared to that of free oleic acid (see Supplementary Fig. 1 for details). The trace amount of free OA can be associated with zinc acetate, but its negative effect was invisible in the investigation. The reaction temperature for investigating the surface chemistry of the precursors was 200 °C to avoid undesired degradation or homogeneous nucleation of the precursors (Supplementary Fig. 2)^42,43^.

**Fig. 1:**
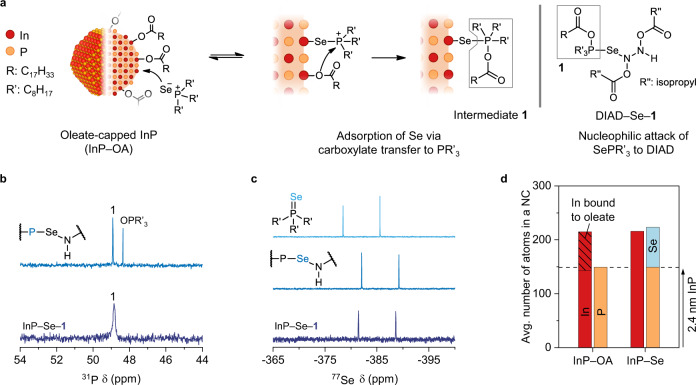
Adsorption of Se from trioctylphosphine selenide to oleate-capped InP NCs. **a** Adsorption pathway of Se to oleate-capped InP NCs (InP–OA) via oleate transfer from the surface to trioctylphosphine selenide (SePR’_3_), yielding oleoyloxytrioctylphosphonium (Intermediate **1**). As shown on the right, this compound can be separately prepared by the nucleophilic addition of SePR’_3_ to diisopropyl azocarboxylate (DIAD) in the form of DIAD–Se–**1**. **b** ^31^P NMR spectra of InP–Se–**1** (deep blue) and oleoyloxytrioctylphosphonium–DIAD selenide (DIAD–Se–**1**, blue). **c** ^77^Se NMR spectra of InP–Se–**1** (deep blue), DIAD–Se–**1** (blue), and SePR’_3_ (light blue). **d** Average number of In (red), P (orange) and Se (light blue) atoms per InP NC before (InP–OA) and after the addition of SePR’_3_ (InP–Se). The hatched region in InP–OA represents the number of Inbound to the oleate ligand. The dashed line indicates the average number of P per InP–OA. Source data are provided as a Source Data file.

The reaction between SePR’_3_ and InP–OA (hereinafter, InP–Se) generated oleoyloxytrioctylphosphonium [R’_3_(OCOR)P^+^; R is oleyl; Intermediate **1**] bound to the Se adatom (InP–Se–**1)** via nucleophilic attack of an oleate ligand on SePR’_3_ (left in Fig. 1a). Intermediate **1** had a downfield-shifted peak at 48.8 ppm in the ^31^P NMR spectrum (deep blue in Fig. 1b). The additional P–O and remaining P–Se linkages strongly attract the electron cloud surrounding P; therefore, its characteristic peak downshifted from OPR’_3_, containing a single P=O linkage. ^1^H diffusion-ordered spectroscopy (DOSY) of InP–Se revealed that Intermediate **1** did not dissociate from the NCs’ surface. The diffusion coefficient (*D*) and corresponding hydrodynamic radius of the oleate-including species were comparable to those of InP–OA, and no side products with a smaller *D* were observed (see Supplementary Fig. 3 for details). The slightly broadened ^31^P NMR signal and invisible ^31^P–^77^Se coupling ( *J*_P–Se_) of **1** also reflected its surface binding, which was attributed to transversal interproton dipolar relaxation in the crowded environment^44^. Nevertheless, the linewidth broadening in InP–Se–**1** was significantly smaller than that of other P-based ligands on the NCs’ surface^44^. This implies that the linkage Se–**1** is a well-defined Se–P bond, thus differing from the surface binding of P-based ligands that experiences a complicated chemical environment of adatoms. The upfield shift of the doublet peaks of InP–Se–**1** from SePR’_3_ in the ^77^Se NMR spectrum (deep blue in Fig. 1c) was attributed to the modified linkage from Se=P to Se–P, which retained more electron clouds at Se and consequently enhanced shielding. The formation of **1** via nucleophilic attack of the oleate to SePR’_3_ was also supported by the amount of Se adatoms in InP–Se. The number of Se adatoms on InP–Se (74, red in Fig. 1d and Supplementary Table S1) concurred with the number of oleates on InP–OA (72, hatched region of InP–OA in Fig. 1d; details in Supplementary Table S1 and the “Methods” section).

To elucidate the origin of the ^31^P chemical shift (δ) of InP–Se–**1**, a similar compound was separately prepared using diisopropyl azocarboxylate (DIAD) (Fig. 1a, right). The electron-accepting diazenyl group in DIAD allowed Se to form a linkage with N to produce a betaine compound containing P^+^. Nucleophilic attack of the deprotonated oleic acid on P^+^ yielded **1** bound to DIAD–Se (DIAD–Se–**1**; Supplementary Fig. 4 and Supplementary Note 1). DIAD–Se–**1** displayed an identical ^31^P δ to InP–Se at 48.8 ppm (blue in Fig. 1b), thus confirming the presence of a P–O linkage in **1**. The minute difference between the ^77^Se NMR spectra of the two compounds was attributed to the different chemical environments, and the DIAD backbone supplied a higher electron density to Se. Intermediate **1** is known as a key byproduct in the reaction between metal carboxylate and trialkylphosphine chalcogenide; this was previously inferred from its bypass reaction with alcohol because of its labile nature^20^. In this study, however, the chemical identity of Intermediate **1** was directly observed owing to the stabilisation through chemical bonding with Se on InP–Se or in DIAD–Se and the large steric hindrance associated with one oleate and three octyl groups.

For the reaction of Zn(OA)_2_ with InP NCs (hereinafter, InP–Zn), surface-exclusive In-to-Zn exchange was observed on the surface, and the gradual blueshift of the absorption spectra indicated a reduced effective size of InP NCs through In-to-Zn exchange on the surface (Supplementary Fig. 5a)^45–47^. This reaction caused the covalently bound oleate ligands to migrate from Zn(OA)_2_ to the surface. InP–Zn prepared using stearate-capped InP NCs led to the emergence of a methine peak in the ^1^H NMR spectrum (Supplementary Fig. 5b).

**Fig. 2:**
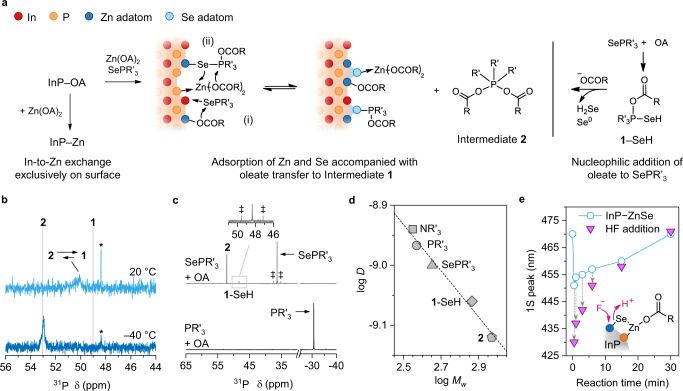
Formation of Zn–Se linkage on oleate-capped InP NCs. **a** Introduction of Zn from zinc oleate [Zn(OA)_2_] to InP–OA through the In-to-Zn surface exchange (InP–Zn) and formation of Zn–Se linkage on InP–OA in the presence of Zn(OA)_2_ and SePR’_3_ (InP–ZnSe). The latter proceeds together with adsorbed species between (i) an exchanged oleate-capped Zn adatom and datively bound SePR’_3_ or (ii) datively bound Zn(OA)_2_ and Intermediate **1** that produces pentacoordinate dioleoyloxytrioctylphosphorane (Intermediate **2**). This compound is separately prepared by nucleophilic addition of oleate to SePR’_3_, as shown on the right. **b** ^31^P NMR spectra of InP–ZnSe (reacted at 200 °C) recorded at 25 °C (top, light blue) and –40 °C (bottom, blue). Asterisk (*) indicates OPR’_3_. **c** ^31^P NMR spectra of reaction products using SePR’_3_ and OA (top, grey), and PR’_3_ and OA (bottom, black). Double dagger (‡) represents ^31^P–^77^Se coupling (*J*_P–Se_ = 680 Hz). **d** Diffusion coefficient (*D*)–molecular weight (*M*_w_) plot of trioctylamine (NR’_3_, square), PR’_3_ (circle), SePR’_3_ (triangle), **1**-SeH (diamond), and **2** (pentagon) characterised by ^1^H diffusion-ordered spectroscopy (DOSY) in CDCl_3_. **e** Temporal change in 1S peak of InP–ZnSe synthesised by the simultaneous addition of Zn(OA)_2_ and SePR’_3_ (light blue circle) to InP–OA NCs with a size of 2.4 nm at 200 °C. The addition of HF in acetone (purple triangle) results in the desorption of adsorbates on InP–ZnSe. Source data are provided as a Source Data file.

The observation of InP–Se–**1** implies that two reaction pathways are responsible for producing Zn–Se bonding on the NCs’ surface when Zn(OA)_2_ and SePR’_3_ are simultaneously introduced into InP–OA (hereafter, InP–ZnSe; Fig. 2a), namely: (i) the reaction of SePR’_3_ with oleate-bound Zn adatoms and (ii) the reaction of Zn(OA)_2_ with Se adatoms. Our study suggests that the former reaction is a transfer of oleate from Zn to SePR’_3_ to produce a Zn–Se linkage and **1**. However, the latter requires a different reaction pathway to justify the cleavage of one of the Zn–O linkages in Zn(OA)_2_, and the formation of Zn–P and Se–Zn–OA linkages. As an extension of (i), pathway (ii) is directed by the formation of pentacoordinate dioleoyloxytrioctylphosphorane [(OCOR’)_2_PR’_3_; Intermediate **2**]; Intermediate **1** accepts an oleate from Zn(OA)_2_ to become unbound **2** and leaves the InP–Se–Zn–OA linkages on the surface. The InP–ZnSe caused the ^31^P NMR peak of **1** to be broadened and deshielded (light blue in Fig. 2b) because the addition of the P–O linkage withdrew the electron cloud from P.

To elucidate ^31^P δ of InP–ZnSe, **2** was separately synthesised using oleic acid and SePR’_3_ at 250 °C (Fig. 2a, right and Supplementary Note 2). In this reaction, a chalcogen anion in ylide^48^ (i.e. ^–^Se–P^+^R’_3_) behaved as a proton acceptor to form oleate from oleic acid. This was corroborated by PR’_3_, which did not react with oleic acid (black in Fig. 2c). The oleate attacked P^+^ in HSe–P^+^R’_3_ to yield **1**–SeH. This unstable compound was decomposed to **1**^+^ and (SeH)^–^ at elevated temperatures, and the nucleophilic attack of oleate on **1**^+^ produced **2**. Intermediate **1**–SeH and **2** exhibited ^31^P δ at 48.4 and 53 ppm, respectively (grey in Fig. 2c), which corresponded to the de-shielding of the O–P–Se and O–P–O linkages. *J*_P–Se_ coupling near 48.4 ppm implied the P–Se linkage was present in **1**–SeH (double dagger in Fig. 2c; Supplementary Note 2 and Supplementary Fig. 6). This identification was further supported by ^1^H DOSY of **1**–SeH and **2**, consistent with the molecular weight (*M*_W_)–*D* relationship referenced to similar compounds (Fig. 2d and Supplementary Fig. 7).

Although **2** was synthesised, its ^31^P NMR spectrum was not identical to that of InP–ZnSe at room temperature. However, at –40 °C, InP–ZnSe exhibited a downfield shift to ~53 ppm, identical to that of **2** (blue in Fig. 2b), and considerable spectral narrowing. The temperature-dependent spectral narrowing and chemical shift can be explained by the dynamic equilibrium between **1** and **2**^49,50^. This equilibrium is biased to **1** at room temperature and shifts toward **2** as the temperature decreases. Overall, pathway (ii) is in equilibrium between **1** and **2** and completed when **2** is released into the medium.

### Transformation of amorphous ZnSe clusters to epitaxial layer

Observation of chemical equilibrium between intermediates explains the thermodynamic aspects of the pathway (ii). The formation of **2** is virtually an exothermic process translated into partial charge neutralisation of the Se–P linkage to a covalent Se–Zn linkage and **2**^51^. However, both intermediates are crowded with octyl and oleate ligands, so pathway (ii) is hindered by the poor accessibility of the P centre. Although high thermal energy can disentangle this entropic penalty through the decomposition of the intermediates to less-crowded OPR’_3_ and byproducts, the reaction at 200 °C is barely capable of dispelling this barrier. As shown in Supplementary Fig. 8a, InP–ZnSe exhibits saturation of the first exciton (1S) peak redshift due to the steric hindrance of **1** and oleate ligands on the surface. This steric barrier restricts the number of Zn and Se adatoms to that of a single ZnSe epilayer at a given temperature (Supplementary Table S1 and the “Methods” section).

Although the 1S peak redshift of InP–ZnSe was nearly saturated after ~10 min, the PL QY of InP–ZnSe increased monotonously during the reaction time (Supplementary Fig. 8b). Given that PL QY is related to the passivation of surface state on InP NCs^52^, this disparity indicated the presence of an additional reaction bridging the Zn and Se adatoms to yield ZnSe units or clusters after the adsorption (PL QY of InP–Zn and InP–Se are ~1% and ~0%, respectively). The intentional desorption of adatoms using hydrogen fluoride (HF) supported the formation of multiple Zn–Se linkages (Fig. 2e). Fluoride is an X-type ligand that replaces nucleophiles on the surface, similar to oleates or Se–**1**. Its small ionic radius is beneficial for penetrating crowded organic layers composed of **1** and oleates. The introduction of HF to InP–ZnSe confirmed that the 1S peak was blue-shifted by HF, which was a sign of the desorption of Se adatoms, but became invariable as the reaction proceeded (from light blue circle to purple triangle, Fig. 2e). This was attributed to the enhanced resistance of Zn and Se adatoms to HF by the formation of a multidentate ZnSe cluster. The Zn and Se adatoms linked together to form Zn–Se units, and their successive bridging produced a sturdy ZnSe cluster.

**Fig. 3:**
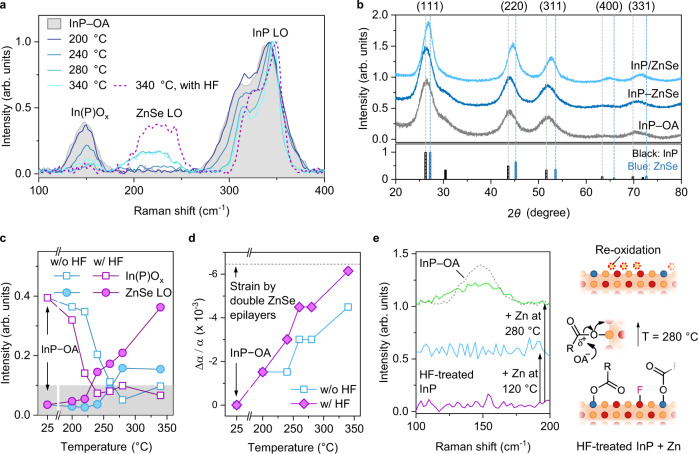
Transformation of Zn and Se adatoms to crystalline ZnSe epitaxial layer. **a** Raman spectra of InP–OA NCs with a size of 3.3 nm (grey) and InP–ZnSe NCs annealed at elevated temperatures of 200 (deep blue), 240 (blue), 280 (light blue), and 340 °C (cyan). InP–ZnSe prepared in the presence of HF (purple dotted line) resulted in a stronger ZnSe LO mode. **b** X-ray diffractograms of InP–OA (grey), InP–ZnSe (blue), and InP/ZnSe (light blue; annealed at 340 °C). For comparison, diffraction lines of bulk InP (black) and ZnSe (blue) are included at the bottom. **c** Intensities of In(P)O_*x*_ (open square) and ZnSe LO phonon modes (solid circle) at different reaction temperatures without (light blue) and with HF (purple). Grey background represents the noise level. **d** Hydrostatic strain (Δ*α*/*α*) applied to InP NCs with a size of 3.3 nm by a single ZnSe epitaxial layer generated at different annealing temperatures with (purple solid diamond) and without HF (light blue open square). The dashed line represents theoretical compressive strain by double ZnSe epitaxial layers based on the elastic continuum theory. **e** Raman spectra of InP–OA at ~150 cm^–1^ with sequential treatments: HF addition at 120 °C (purple), and Zn(OA)_2_ addition at 120 °C (light blue) and at 280 °C (green). Dotted grey line indicates pristine InP–OA. All Raman spectra were normalised to the InP LO peak. The surface migration of oleate and the proposed oxidation pathway are illustrated on the right. All samples are characterised using InP–OA NCs with a size of 3.3 nm. Source data are provided as a Source Data file.

The structural identity of the multidentate ZnSe clusters was determined using Raman scattering and XRD. The absence of any characteristic phonon mode (deep blue in Fig. 3a) and invariant XRD diffractogram of InP–ZnSe (blue in Fig. 3b) indicates that the multidentate ZnSe clusters have an amorphous macromolecular structure. However, as InP–ZnSe aged at elevated temperatures, the broad ZnSe longitudinal optical (LO) phonon (at ~220 cm^–1^)^53^ became visible over 260 °C, and simultaneously, the InP LO phonon mode was blue-shifted (from deep blue to cyan, Fig. 3a). The trend of the XRD peak shift to a higher angle (light blue, Fig. 3b) can be translated into the transformation of amorphous ZnSe to a crystalline ZnSe epilayer coherent to the underlying InP NCs (hereinafter, InP/ZnSe). The compressive strain by the ZnSe epilayer shrunk the lattice of InP NCs, which induced a blue-shift of the InP LO phonon^54^ and the XRD peak shift to a higher angle.

The emergence of ZnSe LO phonon mode at elevated temperatures disclosed the presence of an energetic barrier corresponding to imparting the crystallinity and coherency to amorphous ZnSe, which has been neglected in typical heteroepitaxy schemes executed at high temperatures (typically, ≥300 °C). This barrier might be composed of various elementary reactions associated with the decomposition and release of intermediate species, formation of tetrahedral connections between Zn and Se, coherent interface formation between the underlying surface and overgrown layers, and so on. In the following investigation, the surface oxides was found to be one of the main culprits retarding epilayer formation and, more importantly, influencing the uniformity of the epilayer.

### Re-oxidation of InP surface by zinc carboxylate

Surface oxides on InP NCs were known to be introduced by the trace amount of water released by the ketonization of free carboxylic acids.^39,55^ The InP–OA used in this study was also covered with surface oxides; a comparison of the Raman spectrum with that of oxide-free InP NCs assigned the peak at ~150 cm^–1^ as In(P)O_*x*_ and the sideband broadening of InP LO (centred at ~350 cm^–1^) at approximately 290 and 370 cm^–1^ for In_2_O_3_^56^ (Supplementary Fig. 9a). Additionally, (In)PO_*x*_ and hydroxide were detected at ~132 and ~531.2 eV, respectively, by X-ray photoelectron spectroscopy (XPS) (Supplementary Fig. 9b, c). These investigations classified the oxygen species on InP–OA into two categories: carboxylate and hydroxide as *X*-type surface ligands and In(P)O_*x*_ exclusively visible in Raman scattering. In(P)O_*x*_ became invisible in the Raman spectra as the temperature increased to 340 °C, resulting from the replacement of In–O and P–O linkages with In–Se and P–Zn linkages, respectively (Fig. 3a). Because the oleate ligands were easily replaced by Se–**1** at 200 °C (Fig. 1d), hydroxide and InPO_*x*_ appeared to be the main constituents that elevated the energetic barrier in the ZnSe epilayer formation.

HF is known as an etchant for surface oxides and has recently been employed in the heteroepitaxy of ZnSe on InP NCs to achieve an oxide-free interface^30^. Moderate amounts of HF at 120 °C eliminated the majority of In(P)O_*x*_ (Supplementary Fig. 10d, e) and replaced a considerable portion of carboxylate and hydroxide with fluoride^55^ (Supplementary Fig. 10a–c). This oxide removal immediately improved the quality of the ZnSe epitaxial layer compared to pristine InP–OA; InP/ZnSe NCs using HF-treated InP NCs exhibited higher PL QYs (from ~50% to ~70%; Supplementary Fig. 11d) and a stronger ZnSe LO mode (purple dotted line in Fig. 3a; see Supplementary Fig. 12 for details). The HF-treated InP NCs reduced the onset of In(P)O_*x*_ decrement and ZnSe LO increment by ~20 °C during the growth of the ZnSe epilayer (Fig. 3c). Moreover, the ZnSe epilayer on HF-treated InP NCs displayed improved coherency. The calculation of compressive strain (Δ*α*/*α*) by two ZnSe epilayers on 3.3-nm InP NCs^57^ agreed well with that of InP/ZnSe based on InP–HF, whereas the counterpart based on pristine InP–OA did not (Fig. 3d; see the “Methods” section for details). This observation concurs with previous knowledge regarding the role of HF and the importance of an oxide-free surface for successful heteroepitaxy^30^. Notably, the fluoride ligands on the HF-treated InP NCs did not participate in the heteroepitaxy reaction. The addition of Zn(OA)_2_ removed fluoride ligands bound to In adatoms via rapid In-to-Zn exchange, as manifested by the blue shift of the 1S peak and removal of the F 1*s* signal in XPS (see Supplementary Fig. 13 for details).

Regardless of the enhanced quality of the ZnSe epilayer after oxide removal, a decrease in the PL QY was observed with increasing temperatures. The PL QYs of the InP/ZnSe NCs were similar at the end of the transformation of the ZnSe epilayer at 340 °C despite HF treatment (Supplementary Fig. 11d). A clue to interpreting this poor PL QY was found in the Raman scattering of InP–Zn; despite successful removal of surface oxide, the In(P)O_*x*_ was recovered during the synthesis of InP–ZnSe at 200 °C (at ~150 cm^–1^ in green spectra; see Supplementary Fig. 12). To elucidate this unknown surface oxidation, the emergence of the oxide signal was investigated for pristine InP–OA and HF-treated InP–OA. First, the reaction of InP–OA with Zn(OA)_2_ at 200 °C generated the signature of (In)PO_4_ in the solid-state ^31^P NMR spectrum (Supplementary Fig. 14). Second, even for HF-treated InP NCs, the In(P)O_x_ peak arose at *T* = 280 °C in the Raman scattering (green in Fig. 3e). These considerations suggest that Zn(OA)_2_ is responsible for this secondary pathway of surface oxidation. The oleate ligands bound to Zn adatoms, introduced by the In-to-Zn cation exchange, were almost immovable at low temperatures, such as 120 °C; therefore, the surface of the NCs remained clean (light blue in Fig. 3e). However, at higher temperatures, the oleate can diffuse out onto the surface and are liable to be captured by In and P adatoms because the bond strength of In–O (bond dissociation energy *E*_In–O_ = 346 ± 30 kJ mol^–1^ at 25 °C) and P–O linkages (*E*_P–O_ = 589 kJ mol^–1^) is stronger than that of Zn–O (*E*_Zn–O_ ≤ 250 kJ mol^–1^)^58^. Although the surface oxidation induced by migrated oleate ligands remains vague, we believe that the nucleophilic attack of another oleate on the carbonyl moiety of the oleate bound to the P adatom is a potential pathway for implanting InPO_x_ on the surface (right in Fig. 3e), which is similar to the generation of OPR’_3_ from Intermediate **1**^59,60^.

### Surface-initiated heteroepitaxy of zinc chalcogenides

The discovery of the chemical sequence of heteroepitaxy and the origin of secondary surface oxidation allowed us to devise a surface-initiated fabrication scheme for core/shell heterostructured NCs featuring a uniform epilayer morphology and defect-free heterointerface. To demonstrate our approach, InP/ZnS NCs were chosen as our test bed; ZnS is a wide band gap material showing reasonable photochemical stability and type-I band gap configuration to InP. However, its lattice mismatch with InP (7.7%) caused the morphology of the ZnS epilayer to be non-uniform, which is prone to lower PL QY of InP/ZnS NCs by nonradiative decay of exciton through the uncovered surface. Therefore, intentionally testing thin ZnS epilayers can be a figure of merit for estimating the efficacy of our devised scheme. We selected Zn(OA)_2_ and SPR’_3_ as precursors for ZnS heteroepitaxy; SPR’_3_ also followed the same surface chemistry as SePR’_3_. Stronger S = P bonding than Se = P merely required a higher reaction temperature of 250 °C to trigger the formation of Intermediate **1** on InP–OA. Without InP–OA, ZnS monomers were not produced under the given conditions (see Supplementary Fig. 15 for further details).

**Fig. 4:**
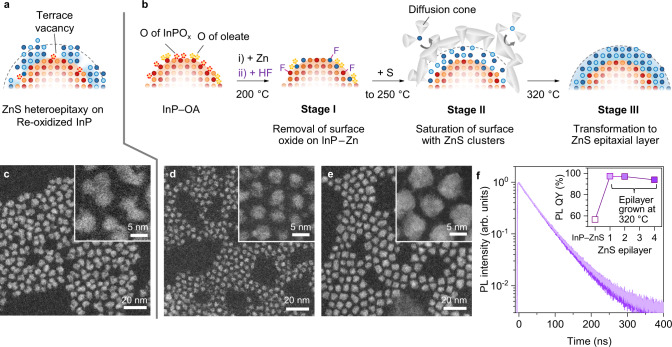
ZnS heteroepitaxy on InP NCs achieving improved uniformity in a single epilayer level. **a** Schematic of conventional ZnS heteroepitaxy performed at high temperatures. Re-oxidation of the surface through Zn(OA)_2_ inevitably introduces unreactive surface oxide, in turn, a non-uniform ZnS epilayer is generated. **b** Proposed fabrication protocol for uniform ZnS epilayer is composed of three stages: Stage I for oxide removal, Stage II for ZnS cluster saturation, and Stage III for ZnS epitaxial layer formation. In Stage II, alkyl chains of **1** and oleate ligand are visualised using grey diffusion cones. For other cases, the diffusion cone was omitted for conciseness. Low and high magnification (inset) dark-field transmission electron microscopy images of InP/ZnS NCs using InP–OA NCs with a size of 3.3 nm: **c** ~4 ZnS epilayers grown on re-oxidised InP NCs; **d** ~1 ZnS epilayer fabricated by (**b**); **e** ~4 ZnS epilayers prepared by (**b**). **f** PL decay dynamics of InP NCs with a size of 3.3 nm coated with different ZnS epilayer thicknesses: ~1 (light purple; size = 3.6 ± 0.4 nm), ~2 (purple; size = 4.7 ± 0.6 nm) and ~4 ZnS epilayers (deep purple; size = 5.6 ± 0.8 nm). Thickness-dependent PL QY values of InP/ZnS NCs are provided in the Inset. Source data are provided as a Source Data file.

In the demonstration of InP/ZnS NCs, HF-treated 3.3-nm InP NCs were adopted as templates. Although typical fabrication schemes starting at high temperatures cannot avoid the secondary oxidation of the surface by Zn(OA)_2_ (Fig. 4a), our protocol passes through additional stages promoting oxide removal and uniform ZnS coverage (Fig. 4b). In Stage I, the In-to-Zn exchange was started at 200 °C and HF treatment followed at 120 °C for oxide removal. The resulting InP–Zn was free from the original and secondary surface oxides and was covered with oleate-capped Zn adatoms. In Stage II, SPR’_3_ was introduced to HF-treated InP–Zn, and the temperature was increased to 250 °C to produce Intermediate **1** via the reaction of metal carboxylate and SPR’_3_. Because Intermediate **1** and oleate ligands block the access of precursors owing to their huge steric hinderance [i.e. ligand cone angle of **1** ≫ 130° for P(CH_2_CH_3_)_3_^61^; Fig. 4b], the ZnS clusters were merely saturated on the surface and barely grew over the second layer irrespective of the amount of precursor (saturation of 1S peak redshift and PL QY; Supplementary Fig. 16a, b). The re-oxidation of the NCs’ surface induced by Zn(OA)_2_ was minimised because the majority of the oleates on the surface had already been replaced or migrated to **1** in the previous stage. In Stage III, which was performed at 320 °C, the ZnS clusters on InP NCs transformed to crystalline ZnS epilayers, and remnant precursors participated in increasing the overall ZnS epilayer thickness, possibly via homogeneous monomer formation and adsorption. The formation of ZnS epilayers was supported in various ways, including redshifted absorption and PL spectra, In(P)O_x_ removal, and increased compressive strain observed in the Raman scattering and XRD (Supplementary Fig. 16c–e).

Although the conventional (i.e. unattended secondary oxidation) and devised schemes led to similar absorption and PL spectra, their morphology exhibited distinct differences; InP NCs contaminated with residual oxide produced irregular ZnS epilayers with terrace vacancies (Fig. 4c), our proposed scheme realised highly uniform ZnS epilayers (Fig. 4d, e and Supplementary Fig. 17). Notably, a single ZnS epilayer (thickness of ~0.3 nm; composed of a Zn layer introduced by In-to-Zn exchange and an S layer grown on the surface) achieved near unity PL QY (97.3%) with monoexponential PL decay (rate constant of ~0.023 ns^–1^). Such superb optical properties were sustained up to a thickness of ~1.2 nm (~4 ZnS epilayers) (Fig. 4f), which verified that the formation of defectless and conformal ZnS epitaxial layers is possible through careful design of the heteroepitaxy process. Although the uniform ZnS epilayer improved the photostability under ultraviolet light exposure compared to its counterpart, the extent of the improvement was marginal (Supplementary Fig. 18). Insufficient electron confinement of thin ZnS epilayers is thought to make the PL QY susceptible to surface chemistry alterations, such as ligand desorption and oxidation of ZnS surface. We believe that a thicker ZnS epilayer that ensures complete carrier confinement is required for practical applications demanding high photostability.

## Discussion

In summary, we have shed light on the heteroepitaxial chemistry of zinc chalcogenides on InP NCs using metal carboxylate and trialkylphosphine chalcogenides as precursors. Multilateral NMR analysis elucidated the molecular structure of the intermediates and metal ion adsorption sequence, which proceeded through the transfer of carboxylate ligands to trialkylphosphine to produce acyloxytrialkylphosphonium and diacyloxytrialkylphosphorane. Our microscopic approach to the heteroepitaxy process aided us in finding the secondary surface oxidation pathway of InP NCs by metal carboxylate and understanding the influence of oxidised NCs’ surfaces on epilayer uniformity. The high affinity of group V elements to oxygen^58^ and the consequent liability to oxidation can explain why III–V/II–VI heterostructured NCs have taken a long time to achieve the quality of II–VI/II–VI NCs despite their similar origins. Based on a profound understanding of the heteroepitaxy mechanism on the surface of colloidal NCs, we devised a heteroepitaxy scheme for core/shell heterostructured NCs. For the most lattice-mismatched ZnS against InP, we could realise uniform and interfacial defect-free single ZnS epilayer (~0.3 nm thick) on InP nanocrystals, confirmed by a photoluminescence quantum yield of 97.3%. We believe that an understanding of the surface chemistry of heteroepitaxy is crucial for exploring synthesis schemes for multifunctional and versatile material libraries made up of heterostructured NCs.

## Methods

### Materials

Indium acetate [In(ac)_3_, 99.99%], zinc acetate [Zn(ac)_2_, 99.99%], chloroform-d (CDCl_3_, 99.8%, contains 0.03% TMS), diisopropyl azodicarboxylate (DIAD, 98%), trioctylamine (TOA, 98%), selenium (Se, 99.99%), sulfur (S, 99.98%), tetrahydrofuran (THF, 99.8%), hydrofluoric acid (HF, 48%), and trichloromethane-d_1_ (CDCl_3_, 99.9%, contains 0.5 wt% silver foil as stabiliser) were purchased from Sigma-Aldrich. Oleic acid (OA, 99%), trioctylphosphine (PR’_3_, 97%), stearic acid (99%), nitrosonium tetrafluoroborate (NOBF_4_, 98%), N,N-dimethylformamide (DMF, 99.8%), 1-octadecene (ODE, 99%), and dichloromethand-d_2_ (CD_2_Cl_2_, 99.9%) were purchased from Alfa Aesar. Squalane was purchased from Acros Organics. Tris(trimethylsilyl)phosphine [(TMS)_3_P, 99.9%] was purchased from Uniam. All chemicals were used as received.

### Preparation of reaction precursors

Briefly, 0.5 M of zinc oleate [Zn(OA)_2_] was prepared by reacting 10 mmol of Zn(ac)_2_ and 20 mmol of OA in 13.7 mL of ODE at 120 °C for 24 h at <100 mTorr. For 0.5 M of In(OA)_3_, 10 mmol of In(ac)_3_ and 30 mmol of OA were degassed at 120 °C for 2 h and diluted with ODE to adjust the concentration to 0.5 M. No extra OA was detected by ^1^H NMR. The saturated SePR’_3_ was synthesised by dissolving excess an amount of Se powder (~10% excess to PR’_3_) in PR’_3_ at 200 °C for 24 h under an inert atmosphere. No extra PR’_3_ was detected by ^31^P NMR. The saturated SPR’_3_ was obtained using the same protocol. Free OA and PR’_3_ were excluded from this study to avoid undesired side reactions such as the coordination of PR’_3_ to Zn(OA)_2_^62^, reduction of metal carboxylate by PR’_3_^63^ or dissolution of InP by OA^64^.

### Synthesis of InP NCs

All procedures were executed under an inert atmosphere. Briefly, 4 mL of In(OA)_3_ and 10 mL of ODE was placed in a round-bottom flask and degassed at 110 °C for 1 h. After backfilling with N_2_, the temperature was increased to 120 °C and 1 mmol of P(TMS)_3_ diluted with 1 mL of PR’_3_ was rapidly injected. Thereafter, the reaction mixture was heated to 260 °C to initiate the nucleation of InP. The reaction temperature was maintained until the first exciton peak of InP NCs reached ~470 nm, corresponding to a diameter of 2.4 nm^65^. To synthesise InP NCs with a diameter of 3.3 nm, additional In and P precursors were introduced until the first excitonic peak reached ~580 nm^62,66^. Stearic acid was used instead of OA to synthesise stearate-capped InP NCs. The as-synthesised InP NCs were purified twice using the typical precipitation (acetone)/redispersion (toluene) method and redispersed in toluene for storage.

### Synthesis of InP–Se, InP–Zn and InP–ZnSe NCs

All reactions were executed under an inert atmosphere. Briefly, 5 mL of ODE and 220 nmol of InP NC were placed in a 50-mL round-bottom flask and degassed at 120 °C for 30 min. After backfilling with N_2_ and increasing the temperature to 200 °C, 0.16 mmol of SePR’_3_ or/and 0.25 mmol of Zn(OA)_2_ was introduced into the core solution and reacted for 30 min to obtain Se-treated (InP–Se), Zn-treated (InP–Zn), and Zn and Se-treated InP NCs (InP–ZnSe). The overall reaction volume was maintained at 5 mL. To prepare InP–ZnSe using 3.3-nm InP NCs, 220 nmol of InP NCs was reacted with 0.40 mmol of SePR’_3_ and 0.50 mmol of Zn(OA)_2_ at 200 °C. The number of precursors corresponded to two ZnSe epitaxial layers equivalent to twice the (111)-plane spacing of InP. InP–Zn and InP–ZnSe were purified five times using the precipitation (acetone)/redispersion (toluene) procedure. InP–Se was purified twice to prevent undesired precipitation because of the dative nature of the oleoyloxytrioctylphosphonium ligand.

### Synthesis of the oleoyloxyphosphonium cation

The reaction procedure reported by Jenkins et al.^59,60^ was modified. The reaction and sampling were executed in an N_2_-filled glovebox (O_2_, H_2_O < 0.1 ppm). Prior to use, OA and PR’_3_ were degassed under vacuum (~100 mTorr) at 120 °C and THF was dried overnight using a 3 Å molecular sieve. Thereafter, 0.33 mmol of DIAD, 0.36 mmol of SePR'_3_ and 0.18 mmol of OA were mixed with 3 mL of THF and reacted overnight at room temperature. At the end of the reaction, a small amount of crude solution was mixed with CDCl_3_ for the NMR analysis.

### Synthesis of dioleoyloxytrioctylphosphorane

Briefly, 4 mmol of OA was placed in a round-bottom flask and degassed at 120 °C for 30 min. After increasing the temperature to 300 °C, 4 mmol of SePR’_3_, SPR’_3_ or PR’_3_ was added and reacted for 30 min. Periodically, aliquots were taken from the reactor for reaction intermediates detection. All reactions and samplings were performed under an inert atmosphere.

### Desorption of ZnSe clusters from InP–ZnSe NCs by HF

Separated reaction vessels were prepared, each containing 220 nmol of InP–OA and 5 mL of ODE and degassed at 120 °C for 30 min. After backfilling with N_2_, the temperature was increased to 200 °C, and SePR’_3_ and Zn(OA)_2_ were added to synthesise InP–ZnSe. At reaction times of 0.5, 1, 3, 6, 15, and 30 min, the reaction vessels were rapidly quenched and 0.2 mmol of HF was added to 0.05 mL of acetone at 150 °C to desorb the ZnSe from InP–ZnSe. The desorption occurred immediately after adding the HF. With minimal air contact at room temperature, aliquots were taken from the crude solution and their absorption spectra were recorded.

### Oxide removal on InP–OA NCs

Reported etching procedures with NOBF_4_^67^ were used to remove the oxides on InP–OA. Briefly, 5 mL of NOBF_4_ in 0.05 M of DMF was added to 220 nmol of InP–OA dispersed in 4 mL of octane. After 5 min, the crude solution was precipitated by adding 4 mL of toluene and redispersed in 5 mL of DMF. This sequence was performed twice to completely remove the surface oxides. On completion of the etching process, the InP NCs was purified three times by typical precipitation (toluene)/redispersion (acetone and DMF). All procedures were performed in a glove box to prevent oxidation of the NCs.

### InP/ZnSe heterostructured NCs for Raman spectroscopy

Briefly, 220 nmol of 3.3-nm InP–OA and 10 mL of squalane were placed in a 50-mL round-bottom flask and degassed at 120 °C for 30 min. After backfilling with N_2_ and increasing the temperature to 200 °C, 0.4 mmol of SePR’_3_ and 0.5 mmol of Zn(OA)_2_ were introduced into the reactor and aged for 30 min to obtain InP–ZnSe. To obtain InP/ZnSe NCs annealed at different temperatures, the temperature was increased to 240, 260, 280 or 340 °C and maintained for 20 min. On completion, the reactor was quenched to room temperature and purified five times using acetone and toluene. To obtain HF-treated InP/ZnSe NCs, 0.4 mmol of HF in acetone was introduced into 220 nmol of degassed InP–OA. At 120 °C, the solution was degassed for a further 30 min. The ZnSe heteroepitaxy process on the HF-treated InP NCs was the same as described above.

### HF-treated InP–Zn NCs for Raman spectroscopy

Briefly, 220 nmol of 3.3-nm InP–OA and 10 mL of squalane were placed in a 50-mL round-bottom flask and degassed at 120 °C for 30 min. After backfilling with N_2_, 0.4 mmol of HF dissolved in acetone was added to the reactor and reacted for 30 min under a vacuum. After backfilling with N_2_, 0.5 mmol of Zn(OA)_2_ was added at either 120 or 280 °C.

### InP/ZnS heterostructured NCs

Briefly, 220 nmol of 3.3-nm InP NCs and 10 mL of squalane were placed in a 50-mL round-bottom flask and degassed at 120 °C for 30 min. After backfilling with N_2_, 1.5 mmol of Zn(OA)_2_ was introduced at 200 °C and aged for 10 min. Thereafter, 0.4 mmol of HF was reacted at 120 °C under vacuum to remove surface oxides (Stage I). After backfilling with N_2_, various amounts of SPR’_3_ were introduced and the temperature was increased to 250 °C to saturate the ZnS clusters on the surface (Stage II). To control the ZnS epilayer thickness, the amount of SPR’_3_ was varied as follows: 0.16, 0.4, or 1 mmol for 1, 2, or 4 ZnS epilayers, respectively. The saturation of adsorption was not influenced by the amount of SPR’_3_ added in Stage II, owing to the steric barrier on the surface. Without purification, 2.5 mmol of Zn(OA)_2_ was added and reacted at 320 °C for 10, 20, and 30 min to transform the ZnS clusters to 1, 2, and 4 ZnS epilayers, respectively (Stage III). To obtain InP/ZnS NCs synthesised through the conventional method, 4 mmol of Zn(OA)_2_ was added to the HF-treated InP NCs at 310 °C, followed by 1 mmol of SPR’_3_ (corresponding to 4 ZnS epilayers). SPR’_3_ was injected dropwise for 1 min to prevent homogeneous nucleation of the ZnS NCs.

### Optical characterisation

The absorption spectra of InP NC and its derivatives were collected using an Agilent 8454 UV–visible spectrophotometer. The PL spectra and PL QY were acquired using a HORIBA Fluoromax-4 spectrometer with an integrating sphere module. The PL decay dynamics were measured using a DeltaTime time-correlated single photon counting module integrated with a HORIBA Fluoromax-4 spectrometer. All samples were excited at 402 nm using a laser diode with a resolution of 200 ps and a repetition rate of 1 MHz.

### Raman spectroscopy

Specimens were prepared by depositing dried NCs onto the glass. Raman spectra were recorded using a Thermo DXR2xi instrument. All samples were excited at 455 nm using a laser diode, and the scattering signal was obtained using an electron-multiplying charge-coupled device (CCD). The recorded spectra were fitted with polynomials to subtract the background signal owing to small particle size and photoluminescence.

### Scanning transmission electron microscopy

High-angle annular dark-field images were obtained using a JEOL JEM-ARM200F instrument operating at 200 kV with a cold-field emission gun.

### X-ray diffraction

The X-ray diffractograms of InP–OA, InP–ZnSe, InP–ZnS, InP/ZnSe, and InP/ZnS were acquired using a Rigaku SmartLab diffractometer with a Cu Kα line (*λ* = 1.5418 Å). All samples were purified more than five times through the precipitation/redispersion procedure and dried under a vacuum overnight to obtain a dry powder.

### X-ray photoemission spectroscopy

The XPS spectra and elemental ratios of In, P, and/or Se were measured using a Thermo ESCALAB250Xi instrument with a micro-focusing monochromator and Al Kα radiation (photon energy = 1486.6 eV). All samples were calibrated with C 1*s* peak at 285 eV. The number of In, P, and Se atoms in InP–OA, InP–Se, and InP–ZnSe NCs was calculated from the elemental ratio obtained using XPS.

### Composition analysis of InP–OA, InP–Se, InP–ZnSe, and InP/ZnSe NCs

To compute the average number of In and P atoms per individual InP–OA NCs, we assumed that the size of an InP NC is solely determined by stoichiometric In and P, and not by extra In on the surface. The diameter of pristine InP–OA (1S peak: 470 nm) was determined to be 2.4 nm using the sizing curve^65^. The number of P atoms in a single InP NC can be calculated as 149 for a 2.4 nm InP NCs by the following equation:

1 $$\frac{{V}_{{{{{{\rm{InP}}}}}}\; {{{{{\rm{NC}}}}}}}}{{V}_{{{{{{\rm{InP}}}}}}\; {{{{{\rm{unit}}}}}}\; {{{{{\rm{cell}}}}}}}}\times {N}_{{{{{{\rm{P}}}}}}\; {{{{{\rm{in}}}}}}\; {{{{{\rm{InP}}}}}}\; {{{{{\rm{unit}}}}}}\; {{{{{\rm{cell}}}}}}}$$

where *V*_InP NC_ is the volume of an InP NC, *V*_InP unit cell_ is the volume of an InP unit cell, and *N*_P in InP unit cell_ is the number of P atoms in an InP unit cell. The element ratio of In to P (In:P = 1.44:1; Supplementary Table S1) established the number of In atoms in InP–OA to be 215.

The coverage of the oleate ligand on InP–OA was characterised using ^1^H NMR. We introduced 1000 nmol of ferrocene into 3.5 nmol of InP–OA in 0.6 mL of CDCl_3_ as a concentration reference. The ligand coverage of InP–OA (*Y*) is calculated by the following formula:

2 $$Y=\left(\frac{{A}_{{{{{{\rm{oleate}}}}}}}/2}{{A}_{{{{{{\rm{ferrocene}}}}}}}/10}\right)\left(\frac{F}{N\times S}\right)$$

where *F* is the molar amount of ferrocene, *N* is the molar amount of InP–OA, *S* is the surface area of InP–OA, *A*_oleate_ is the area under the curve in the ^1^H NMR spectra associated with two methine protons in an oleate ligand, and *A*_ferrocene_ is the area under the curve in the ^1^H NMR spectra associated with 10 protons in a ferrocene. For *S* = 18.1 nm^2^ and *A*_ferrocene_/*A*_oleate_ = 19.7, the surface coverage of oleate in InP–OA was 4.0 oleates/nm^2^, which corresponded to 72 oleates per InP NC.

To obtain the nominal amount of Se atoms in a single ZnSe epilayer on a 2.4 nm InP NC, we assumed that (1) the number of P atoms barely changed during the growth of the ZnSe epilayer (149 atoms in pristine InP–OA, Table S1) and (2) the thickness of a single ZnSe epilayer can be regarded as half of the lattice parameter of ZnSe (0.567 nm). The number of Se atoms in a single ZnSe epilayer is calculated as 171 for a 2.4 nm InP NC by the following formula:

3 $$\frac{{V}_{{{{{{\rm{InP}}}}}}/{{{{{\rm{ZnSe}}}}}}\; {{{{{\rm{QD}}}}}}}-{V}_{{{{{{\rm{InP}}}}}}-{{{{{\rm{OA}}}}}}}}{{V}_{{{{{{\rm{ZnSe}}}}}}\; {{{{{\rm{unit}}}}}}\; {{{{{\rm{cell}}}}}}}}\times {N}_{{{{{{\rm{Se}}}}}}\; {{{{{\rm{in}}}}}}\; {{{{{\rm{ZnSe}}}}}}\; {{{{{\rm{unit}}}}}}\; {{{{{\rm{cell}}}}}}}$$

where *V*_InP/ZnSe_ NC is the volume of an InP/ZnSe NC containing a single ZnSe epitaxial layer, *V*_ZnSe_ is the volume of the ZnSe unit cell, and *N*_Se_ in ZnSe unit cell is the number of Se atoms in the ZnSe unit cell.

### Estimation of compressive strain in InP/ZnSe NCs

The deformation of InP NCs by the ZnSe epitaxial layers was calculated by the following equation^68^:

4 $$\frac{\Delta \omega }{\omega }={\left(1+3\frac{\Delta a}{a}\right)}^{-\gamma }-1$$

where *ω* is the LO phonon frequency of pristine InP–OA, Δ*ω* is the change in the LO phonon frequency of InP/ZnSe from that of pristine InP–OA, *a* is the lattice constant of InP, Δ*a* is the lattice parameter change, and *γ* is the Grüneisen parameter (*γ* = 1.24 for InP)^54^.

We assumed that (i) the structure of InP/ZnSe NC was concentric and spherical, (ii) no pressure was applied to the ZnSe epilayer, and (iii) a negligible difference in elastic parameters between InP and ZnSe. The radial strain, Δ*a*/*a*, at the interface is calculated using elastic continuum theory as below^57^:

5 $$\frac{\Delta a}{a}=\frac{2\varepsilon (1-2\nu )}{1-\nu }\frac{(3d+3{d}^{2}+{d}^{3})}{3{(1+d)}^{3}}$$

where *ε* is the relative difference in lattice constants, *ν* is the Poisson ratio of InP, *d* is the reduced radius defined as $d=(R-{r}_{{\rm {c}}})/{r}_{{\rm {c}}}$, *R* is the overall radius, and *r*_c_ is the radius of the InP NCs. We used *ε* = 0.0345, *ν* = 0.36, *R* = 23.5 Å, and *r*_c_ = 16.5 Å to obtain Δ*a*/*a* = –0.00646, where a negative sign represents the compressive strain.

### Nuclear magnetic resonance spectroscopy

^1^H NMR spectra were recorded using a Varian Oxford 300 NMR instrument. ^1^H-decoupled ^31^P NMR spectra were recorded using a Bruker AVANCE III 700 MHz. ^77^Se spectra were recorded using a Bruker Avance III 500 MHz NMR instrument. Variable-temperature ^31^P NMR spectra were recorded using a Bruker Avance III HD 850 MHz Cryo-NMR instrument. ^1^H DOSY spectra were recorded using a Bruker Avance III 600 NMR instrument. CD_2_Cl_2_ was used as the solvent for variable-temperature ^31^P NMR spectroscopy and CDCl_3_ was used for the others. Solid-state ^31^P magic angle spin NMR spectra were recorded using an AVANCE III HD Solid 500 MHz NMR instrument. Resonance frequencies of 300.04, 850.22, 283.56, 202.46, and 95.38 MHz were used for ^1^H, ^1^H DOSY, ^31^P, solid-state ^31^P, and ^77^Se NMR spectral acquisitions, respectively. All samples were prepared in a glovebox, and the NMR spectra were measured within 3 h to prevent degradation or oxidation of the products.

### Statistics and reproducibility

No statistical method was used to predetermine the sample size.

### Reporting summary

Further information on research design is available in the Nature Portfolio Reporting Summary linked to this article.

## Supplementary information

Supplementary Information

Reporting Summary

## Data Availability

All data generated or analysed during this study are included in this published article or in the supplementary information, which is also available from the corresponding author upon request. Source data are provided with this paper.

## Source data

Source Data
