# Supplementary material for: Heteroepitaxial chemistry of zinc chalcogenides on InP nanocrystals for defect-free interfaces with atomic uniformity
Source: Nat Commun. 2023 Jan 3;14:43. doi: 10.1038/s41467-022-35731-2 (PMC9810615; doi:10.1038/s41467-022-35731-2)
Supplement: Supplementary file 1 — Supplementary Information [file 41467_2022_35731_MOESM1_ESM.pdf]

Supplementary Information

Heteroepitaxial chemistry of zinc chalcogenides on InP nanocrystals for defect-free interfaces with atomic uniformity

Yeongho Choi[1,2], Donghyo Hahm[3,4], Wan Ki Bae[3] and Jaehoon Lim[1,2,*]

[1]Department of Energy Science, Centre for Artificial Atoms, Sungkyunkwan University (SKKU), Suwon 16419, Republic of Korea.

[2]SKKU Institute of Energy Science and Technology (SIEST), Sungkyunkwan University, Suwon 16419, Republic of Korea

[3]SKKU Advanced Institute of Nanotechnology (SAINT), Sungkyunkwan University, Suwon, Gyeonggi-do 16419, Republic of Korea.

[4]Chemistry Division, Los Alamos National Laboratory, Los Alamos, New Mexico 87545, United States

* Address correspondence to j.lim@skku.edu

Table of contents

Supplementary Note 1

Supplementary Note 2

Supplementary Figures

Supplementary Fig. 1. Investigation of $Zn(OA)_2$ stock solution.
Supplementary Fig. 2. Stability of $Zn(OA)_2$ and SePR'$_3$ without InP NCs at elevated temperatures.
Supplementary Fig. 3. Inspection on surface bonding of Intermediate 1 on InP–Se.
Supplementary Fig. 4. Synthesis of oleoyloxytrioctylphosphonium.
Supplementary Fig. 5. Surface reaction of $Zn(OA)_2$ with InP–OA.
Supplementary Fig. 6. Synthesis of dioleoyltrioctylphosphorane.
Supplementary Fig. 7. ^{1}H Diffusion-ordered spectroscopy of reaction precursors and intermediates.
Supplementary Fig. 8. Optical properties of InP–ZnSe.
Supplementary Fig. 9. Clarification of surface oxide on pristine InP–OA.
Supplementary Fig. 10. Characterization of HF-treated InP–OA.
Supplementary Fig. 11. Optical properties of InP/ZnSe fabricated using pristine and HF-treated InP–OA NCs.
Supplementary Fig. 12. Raman spectroscopy of InP–ZnSe and InP/ZnSe based on HF-treated InP NCs.
Supplementary Fig. 13. Removal of fluoride from HF-treated InP–OA using $Zn(OA)_2$.
Supplementary Fig. 14. Increment of (In)PO_4 resonance after Zn-carboxylates treatment to InP–OA.
Supplementary Fig. 15. Stability of $Zn(OA)_2$ and SPR'$_3$ without InP NCs and Intermediate 1 formation on InP–OA by SPR'$_3$.
Supplementary Fig. 16. Characterisation of InP/ZnS NCs prepared through the surface-initiated heteroepitaxy process.
Supplementary Fig. 17. Dark-field transmission electron microscopy of InP/ZnS NCs with 2 ZnS epilayers fabricated by the proposed scheme.
Supplementary Fig. 18. Photostability of InP/ZnS NCs.

Supplementary Table

Supplementary Table 1. Chemical composition of the NCs investigated in this study.

Supplementary References

Supplementary Note 1. Characterization of oleoyloxyphosphonium (Intermediate 1).

Acyloxytrialkyl(or aryl)phosphonium is known to be an important intermediate species in the Mitsunobu reaction, which leads to the condensation of carboxylic acid with alcohol[1]. In this reaction, diisopropyl azodicarboxylate (DIAD) is widely employed as a strong electron acceptor and deprotonation agent to produce betaine and carboxylate (i.e. deprotonated carboxylic acid). In this study, DIAD was introduced to exploit its diazenyl moiety as (i) a nucleophile accepting a proton from oleic acid and (ii) an electrophile permitting dative bonding with Se in the single-bonded ylide ($^{-}$Se–P^{+}R'$_3$) to mimic the surface binding of SePR'$_3$ on InP–OA. In our proposed reaction mechanism (Supplementary Fig. 4a), an oleoyloxytrioctylphosphonium cation (Intermediate **1)** bonded to DIAD–Se^{-} (DIAD–Se–**1**) is produced by the nucleophilic attack of oleate on the P atom in betaine. A deficient amount of oleic acid (i.e. oleic acid: SePR'$_3$ = 1:2 molar ratio) was used to prevent the excessive oxidation of Intermediate **1** to trioctyphosphine oxide (OPR'$_3$).

An investigation of ^{31}P and ^{77}Se NMR (orange spectra in Fig. 1b and 1c) revealed the presence and chemical natures of the Se–N and P–Se linkages of DIAD–Se–**1**. The ^{77}Se doublet signal of DIAD–Se–**1** originating from the ^{31}P–^{77}Se coupling ($J_{P\text{–}Se}$ = 680 Hz[2]) shifted upfield compared to that of pristine SePR'$_3$ (yellow in Fig. 1c). We attribute this difference to the formation of a Se–N linkage, in which the electron-rich DIAD backbone contributes to an increase in the electron density surrounding Se. Two distinguishable peaks were observed in the ^{31}P NMR spectrum of DIAD–Se–**1**: a peak at 48.4 ppm attributed to OPR'$_3$ resulting from oxidation of Intermediate **1** and a peak at 48.6 ppm assigned to Intermediate **1**. Because the P atom in DIAD–Se–**1** was bound to two electronegative atoms, Se and O, its chemical shift was downfield from that of SePR'$_3$ (at ~36.4 ppm) and OPR'$_3$. The absence of the^{31}P–^{77}Se coupling can be explained

by retarded molecular diffusion and broadening of the corresponding linewidth. Notably, a proton transfer from the oleic acid to the diazenyl moiety was supplied by the oleate in this reaction; this was substantiated by the upfield-shifted signal from the proton bonded to N in the ^{1}H NMR spectrum of DIAD-Se-1[3] (Supplementary Fig. 2b).

Supplementary Note 2. Characterization of dioleoyloxytrioctylphosphorane (Intermediate 2).

To confirm the chemical identity of the compound using the ^{31}P NMR spectrum of InP–ZnSe, dioleoyloxytrioctylphosphorane (Intermediate **2**) was separately synthesised by the nucleophilic addition of oleate to SePR'$_3$ (Supplementary Fig. 6a). In the proposed reaction process, a chalcogen anion in a singly bonded ylide (e.g. $^{-}$Se–P^{+}R'$_3$)[4] accepts an acidic proton from oleic acid to produce an oleate ligand. This deprotonation reaction derives P^{+} from (hydroseleno)trioctylphosphonium (HSe–P^{+}R'$_3$), which acts as an electrophile. In addition, (hydroseleno)oleoyloxytrioctylphosphorane [or (Z)-1-((hydroselenotrioctylphosphoranyl)oxy)octadec-9-en-1-one (**1**-SeH) is derived by the nucleophilic attack of oleate on HX–P^{+}R'$_3$ In the subsequent stage, the singly bonded P–Se linkage is decomposed into Intermediate **1** and (SeH)$^{-}$. The former, containing electrophilic P^{+}, experiences another nucleophilic attack of oleate to form Intermediate **2**. We suppose that the latter is thermally decomposed into H_2Se, polymeric Se, and elemental Se^{0}.

Two new ^{31}P NMR peaks at 53 ppm and 48.4 ppm were observed after the reaction between SePR'$_3$ and oleic acid at 250 °C (Supplementary Fig. 6b), attributed to Intermediate **2** and Intermediate **1**–SeH, respectively, because of following reasons. First, the P atom in Intermediate **2** was de-shielded to a greater degree by two O atoms than Intermediate **1**–SeH with only one O atom and one SeH. Accordingly, the ^{31}P NMR spectrum of **1**–SeH was shifted slightly upfield with

respect to Intermediate **1** (~0.2 ppm). Second, Intermediate **2** exhibited a single ^{31}P peak, whereas Intermediate **1**–SeH possessed weak satellite peaks with a spacing of 680 Hz which corresponded to the ^{31}P–^{77}Se coupling ($J_{P–Se}$, double dagger in Fig. 2c). Intermediate **2** was also detected when SPR'$_3$ was used instead of SePR'$_3$ (Supplementary Fig. 6c).

Intermediate **1**–SeH appears to be a midway compound in the process yielding Intermediate **2**. Although a decrement of SePR'$_3$ evidently correlates to an increment of Intermediate **2**, the content of Intermediate **1**–SeH was limited to a mere ~2% over the entire reaction period (Supplementary Fig. 4d). As the reaction proceeded, gradual yellowing of the reaction medium was observed, followed by the formation of grey precipitate characterised as Se^0 (Supplementary Fig. 6e). In addition, a trace amount of PR'$_3$ was only detected in the initial stage (at 10 min; Supplementary Fig. 6b). This observation suggests that Intermediate **2** was converted by the decomposition of **1**–SeH into $\mathbf{1}^+$ and $(SeH)^-$, followed by a nucleophilic attack of oleate to $\mathbf{1}^+$, during which an additional oleate was supplied from $\mathbf{1}^+$. Simultaneously, $(SeH)^-$ became a polymeric Se, as predicted by the yellowish solution[5] and precipitated Se^0. The PR'$_3$ released by **1**–SeH was recovered as SePR'$_3$ by the reaction with Se^0. The premise was further supported by the determination of their molecular weight (M_W) using ^1H diffusion ordered spectroscopy (DOSY), referenced from the extrapolation of the relationship between M_W and the diffusion coefficient (D) using similar compounds (Fig. 2d and Supplementary Fig. 7).

Supplementary Figures

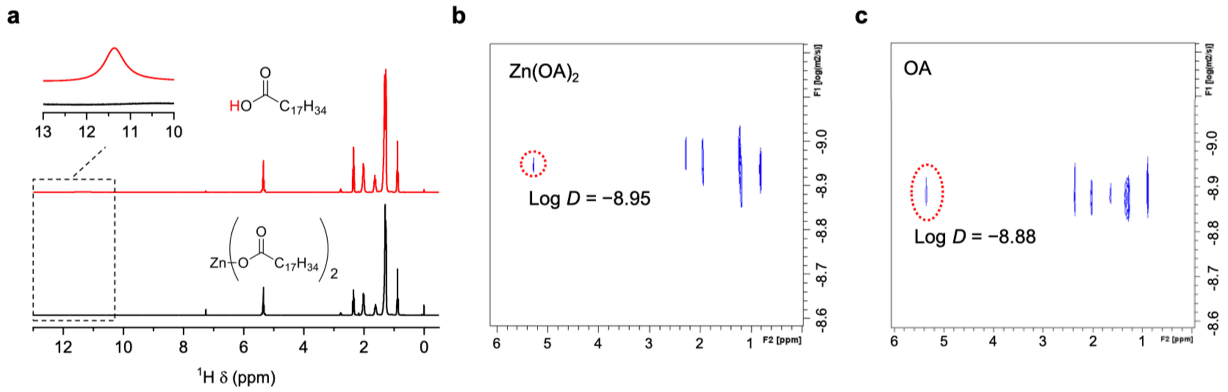

Supplementary Fig. 1| Investigation of $Zn(OA)_2$ stock solution. a, Comparison of ^{1}H NMR spectra of $Zn(OA)_2$ (bottom) and oleic acid (top). Inset magnifies the chemical shift corresponding to the acidic proton of carboxylic acid (magnification: ×100). ^{1}H DOSY spectra of **b**, $Zn(OA)_2$ and **c**, free oleic acid, where the red dotted circle indicates the resonance peak from methine protons. Free oleic acid and macromolecular complexes were not detectable in the $Zn(OA)_2$ stock solution. We note that a trace amount of free oleic acid undetectable by ^{1}H DOSY and NMR can be remained in the $Zn(OA)_2$ stock solution while its negative effect was invisible in the investigation.

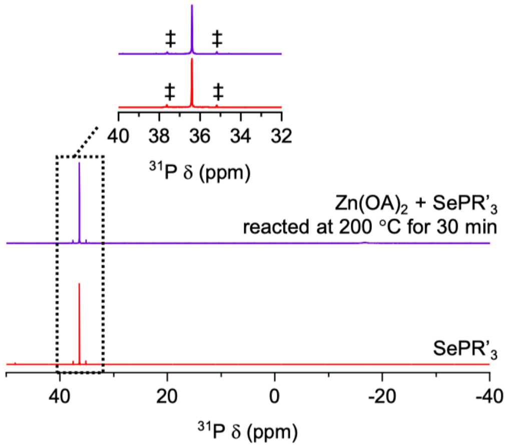

Supplementary Fig. 2| Stability of $Zn(OA)_2$ and SePR'$_3$ without InP NCs at elevated temperatures. ^{31}P NMR spectra of pristine SePR'$_3$ (bottom) and a mixture of 0.2 mmol of SePR'$_3$ and 0.4 mmol of $Zn(OA)_2$ in 5 mL of ODE reacted at 200 °C for 30 min (top). The double dagger indicates ^{31}P–^{77}Se coupling ($J_{P\text{–}Se}$ = 680 Hz). The absence of by-products (e.g., O=PR'$_3$) and the invariable chemical shift of SePR'$_3$ supports the premise that no reaction took place under this condition.

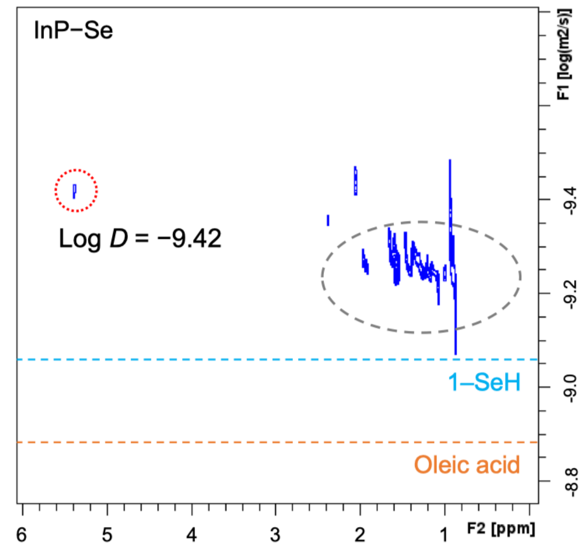

Supplementary Fig. 3| Inspection on surface bonding of Intermediate 1 on InP–Se. ^{1}H DOSY spectrum of InP–Se. Squalane ($C_{30}H_{62}$, grey dashed circle) was used as the solvent to avoid spectral overlap of the methine protons of Intermediate **1** (red dotted circle) and 1-octadecene. The diameter of InP–Se, calculated from the Debye-Einstein equation, was 2.3 nm. No trace of free Intermediate **1** (cyan dashed line; Log *D* = –9.06) or free oleic acid (orange dashed line; Log *D* = –8.88) attests that Intermediate **1** is bonded to the NCs' surface.

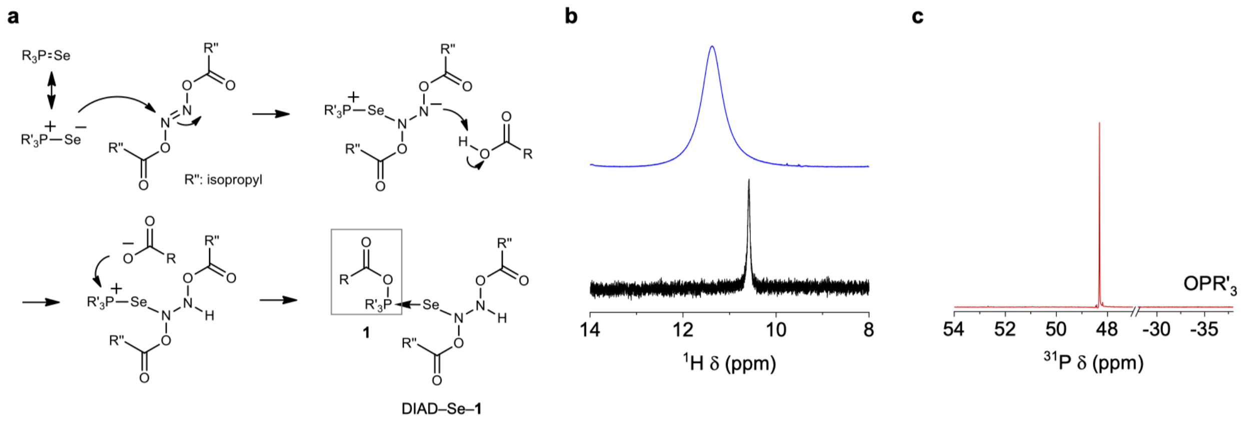

Supplementary Fig. 4| Synthesis of oleoyloxytrioctylphosphonium. a, Proposed reaction mechanism producing **1** via nucleophilic addition of SePR'$_3$ to the diazenyl group in diisopropyl azocarboxylate (DIAD). **b,** ^{1}H NMR spectra of oleic acid (top) and oleoyloxytrioctylphosphonium DIAD selenide (bottom) in a down-field region. The proton bound to carboxylate group migrated to the diazenyl group. **c,** ^{31}P NMR spectrum of pure OPR'$_3$.

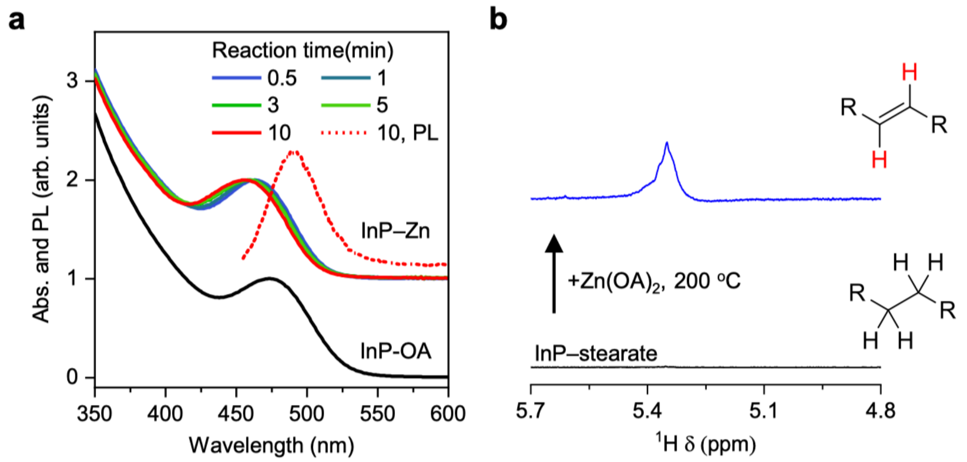

Supplementary Fig. 5| Surface reaction of $Zn(OA)_2$ with InP–OA. a, Absorption spectra of pristine InP–OA (black) and InP–Zn reacted at 200 °C as a function of time (InP–Zn; coloured): 0.5 (blue), 1 (sky blue), 3 (deep green), 5 (yellowish green), and 10 min (red). The PL spectrum of InP–Zn at 10 min is included as red dotted line. **b**, ^1H NMR spectra of stearate-capped InP (InP–stearate, bottom) and $Zn(OA)_2$-treated InP–stearate at 200 °C for 30 min (top). The In-to-Zn exchange with $Zn(OA)_2$ left oleate ligands bound on the surface InP NCs, as demonstrated by the broadened methine peak.

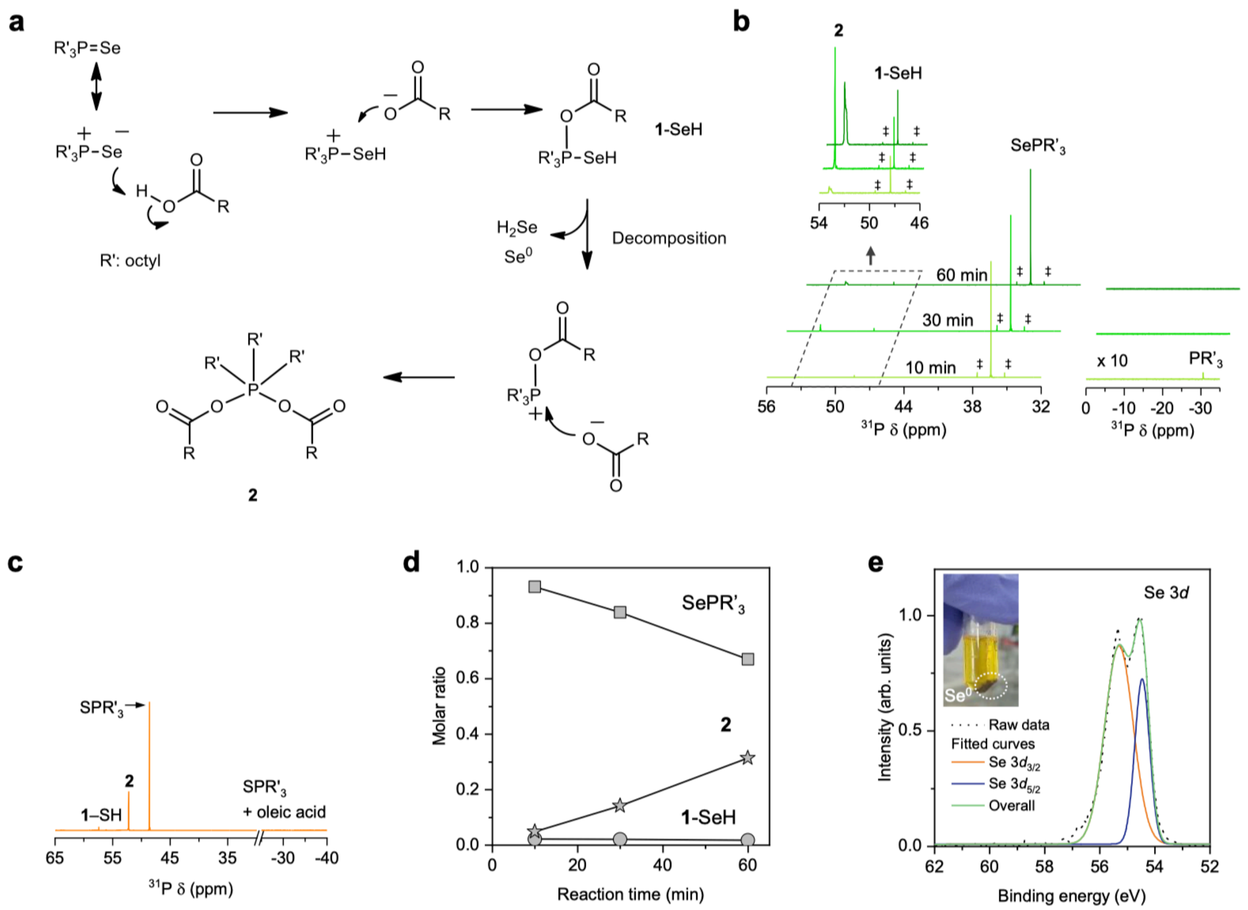

Supplementary Fig. 6| Synthesis of dioleoyltrioctylphosphorane. **a,** Proposed reaction mechanism with nucleophilic addition of oleate to SePR'$_3$, yielding **1**–SeH and **2**. **b,** The time-dependent ^{31}P NMR spectra of the reaction products between SePR'$_3$ and oleic acid at 250 °C. The inset magnifies the spectral range from 46 to 54 ppm to show **2** and **1**–SeH in detail. The double dagger indicates $J_{P–Se}$ coupling. **c**, The ^{31}P NMR spectrum of the reaction product between SPR'$_3$ and oleic acid. **d**, Relative concentrations of SePR'$_3$ (square), **1**–SeH (diamond), and **2** (star) as a function of time, acquired from their integrals in **b**. **e**, Se 3*d* electron binding energy of the precipitate obtained from the crude reaction product of SePR'$_3$ and oleic acid. Inset is a photograph of the crude reaction product after centrifugation at 12,225 × g for 5 min. Precipitated elemental Se is indicated by a dotted circle.

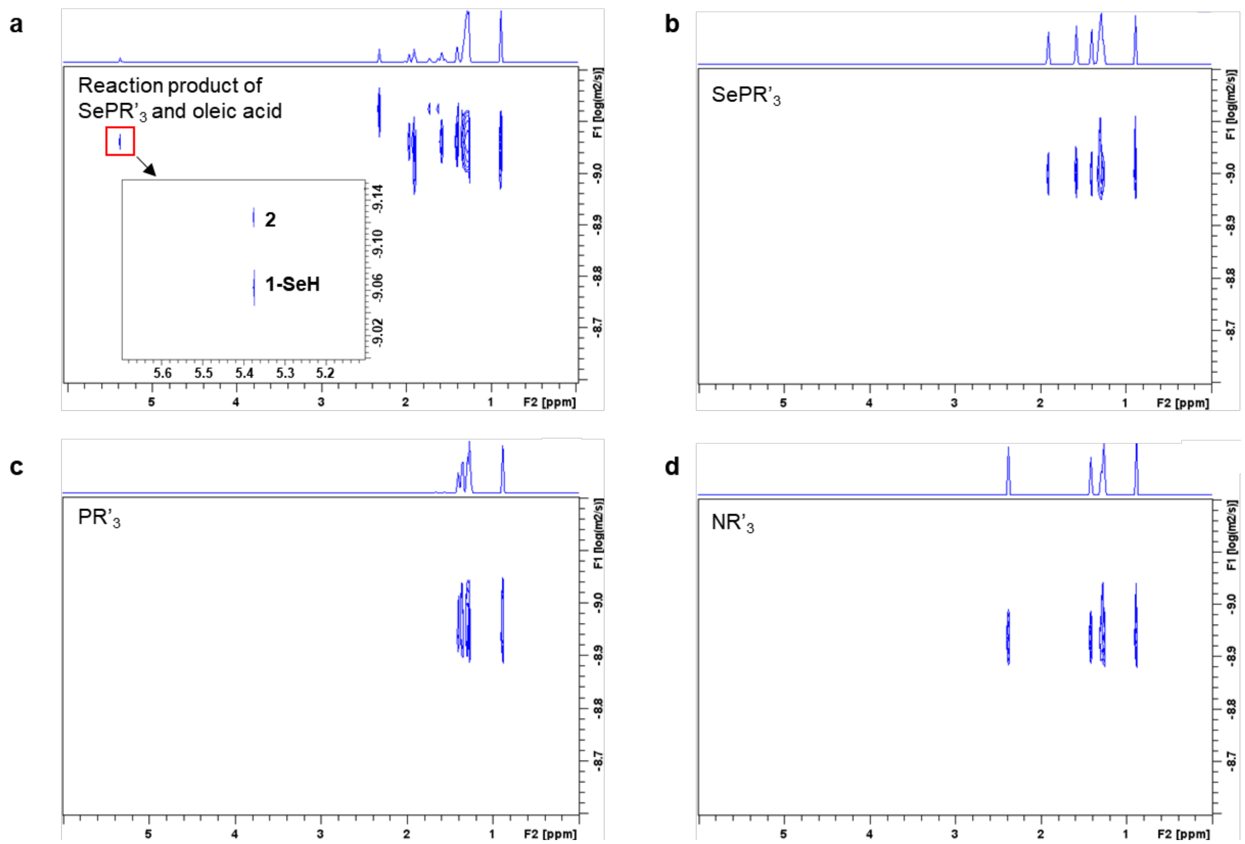

Supplementary Fig. 7| ¹H Diffusion-ordered spectroscopy of reaction precursors and intermediates. ¹H diffusion-ordered spectroscopy of **a**, the reaction product of PR'$_3$ and oleic acid (a mixture of **1**–SeH, **2**, etc.), **b,** SePR'$_3$, **c**, PR'$_3$, and **d**, n-trioctylamine (NR'$_3$) in $CDCl_3$. Inset in **a** magnifies the methine protons originating from **2** and **1**–SeH.

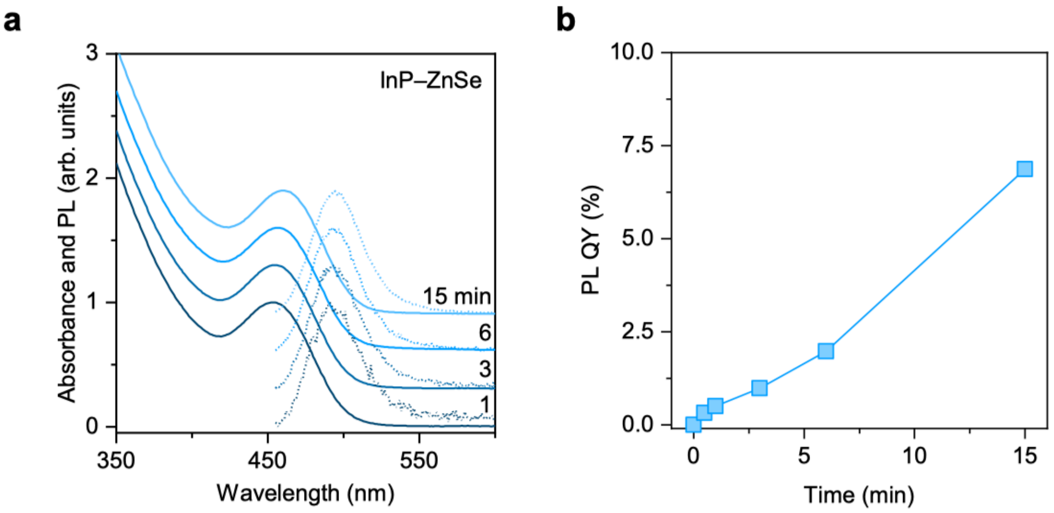

Supplementary Fig. 8| Optical properties of InP–ZnSe. a, Absorption (solid) and photoluminescence (PL) spectra (dotted line) of InP–ZnSe using InP–OA with a size of 2.4 nm as a function of time. $Zn(OA)_2$ and $SePR'_3$ were simultaneously added to InP–OA at 200 °C. **b**, PL quantum yield (QY) of InP–ZnSe reacted at 200 °C as a function of time.

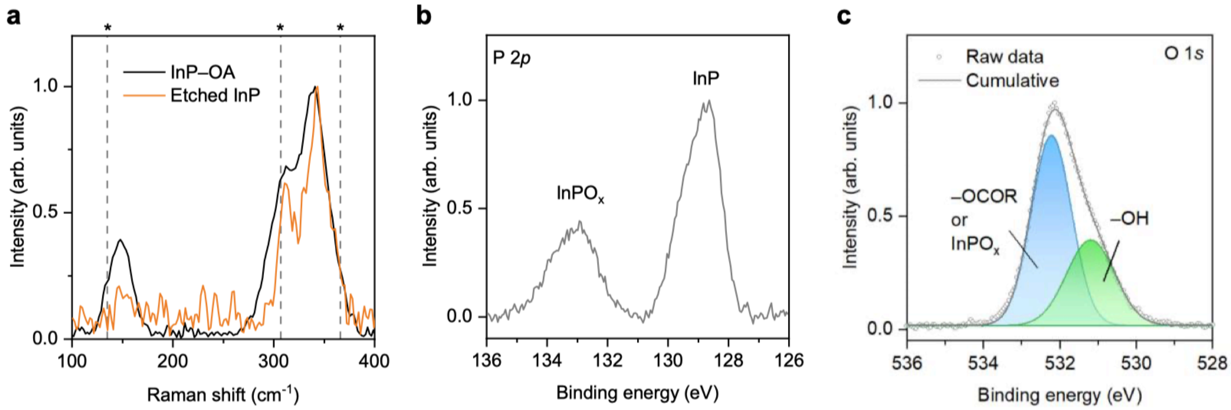

Supplementary Fig. 9| Clarification of surface oxide on pristine InP–OA. a, Raman spectra of pristine InP–OA (black) and InP–NCs etched twice with an excess amount of $NOBF_4$ (orange). The dashed grey lines, indicated using an asterisk, at 135, 307, and 366 cm^{-1} indicate the vibration modes of bulk In_2O_3.[6] After the etching process, signals from the oxide are diminished. **b**, P 2*p* electron binding energy of InP–OA. **c**, O 1*s* electron binding energy of InP–OA, where carboxylate (blue), $InPO_x$ (blue), and hydroxide (green) are detected.

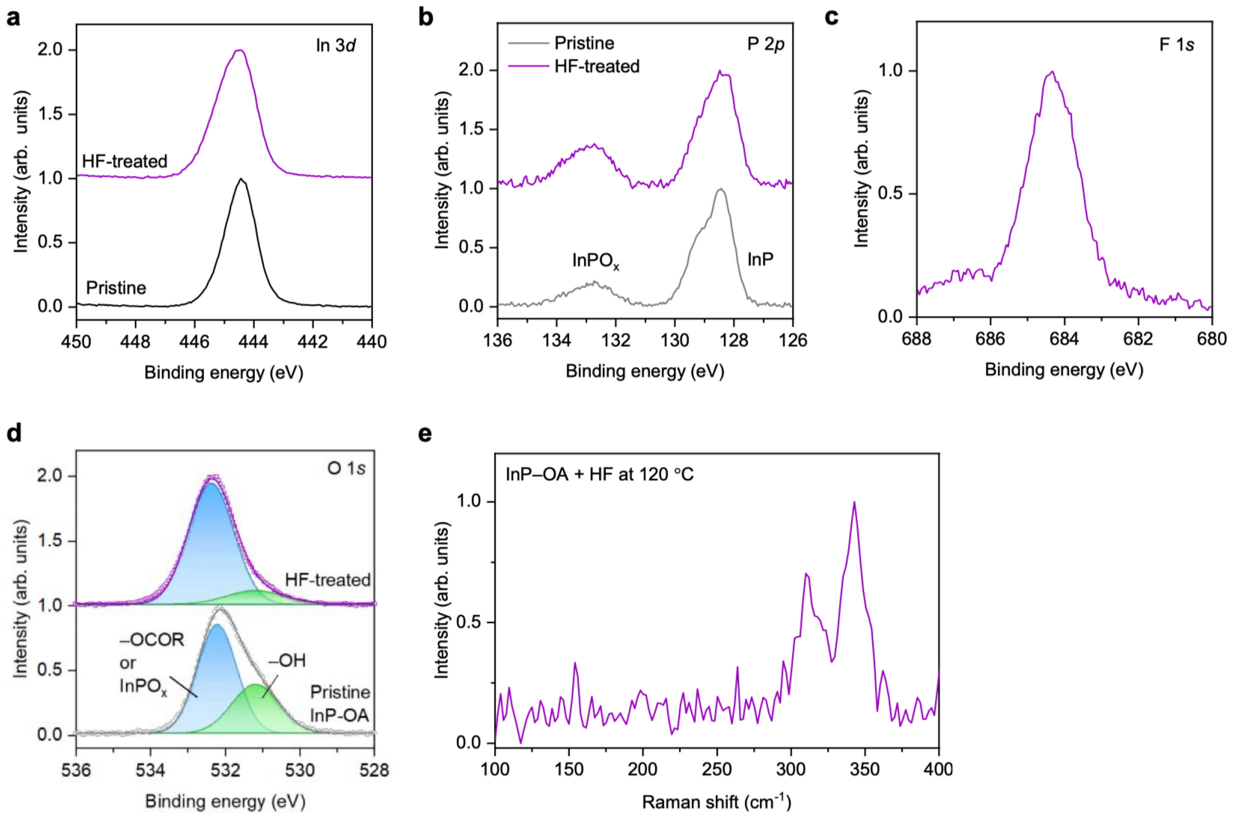

Supplementary Fig. 10| Characterization of HF-treated InP–OA. a, In 3*d*, **b**, P 2*p* region of pristine InP–OA (grey) and HF-treated InP–OA (purple). For comparison, InP–OA is included as grey solid line. **c**, F 1*s* region of HF-treated InP–OA. **d**, O 1*s* region of InP–OA (grey) and InP–HF (purple). Open symbols express the experimental data and solid lines the Gaussian fit for the hydroxyl group (at 531.2 eV, green), carboxylate, and InPO$_x$ (at 532.2 eV, blue), and their cumulative fit. **e**, Raman spectrum of HF-treated InP–OA, where the phonon mode of In_2O_3 was diminished. The diameter of InP–OA applied to these characterisations is 3.3 nm.

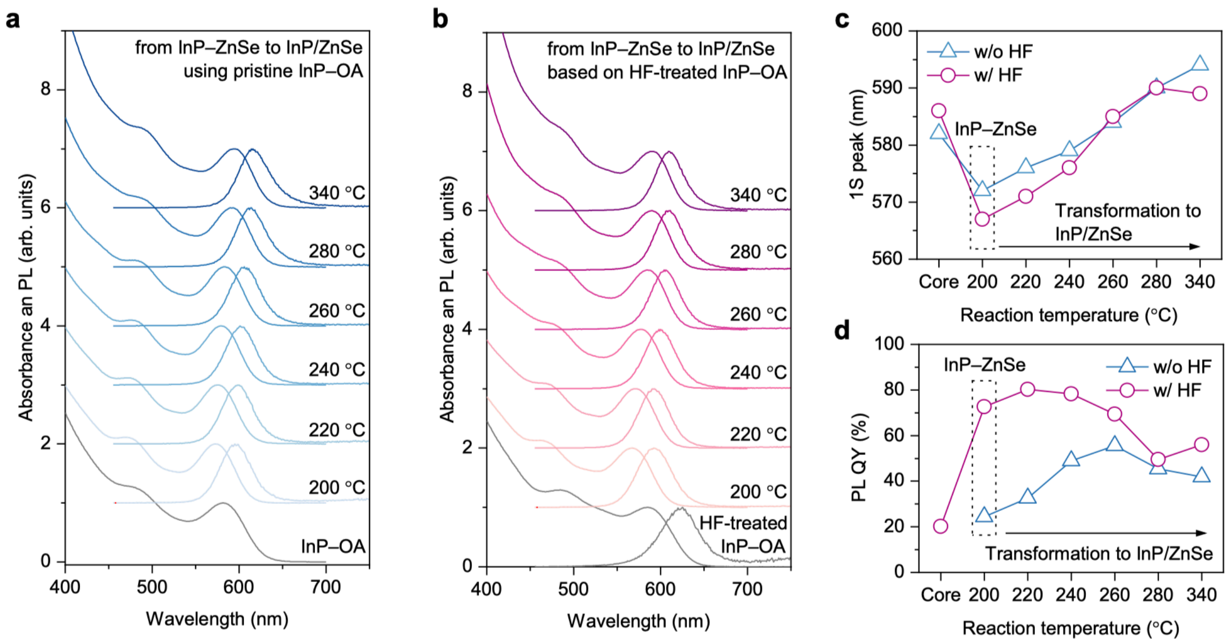

Supplementary Fig. 11| Optical properties of InP/ZnSe fabricated using pristine and HF-treated InP–OA NCs. Absorption and PL spectra of InP–ZnSe and InP/ZnSe NCs taken at different reaction temperatures using **a**, pristine and **b**, HF-treated InP–OA NCs. The size of the original InP–OA was 3.3 nm. **c**, 1S peak and **d**, PL QY of InP–ZnSe at 200 °C, and the InP/ZnSe NCs transformed at increasing temperatures: the sky blue triangle and purple circle for the heterostructured NCs based on the original and HF-treated InP–OA NCs, respectively.

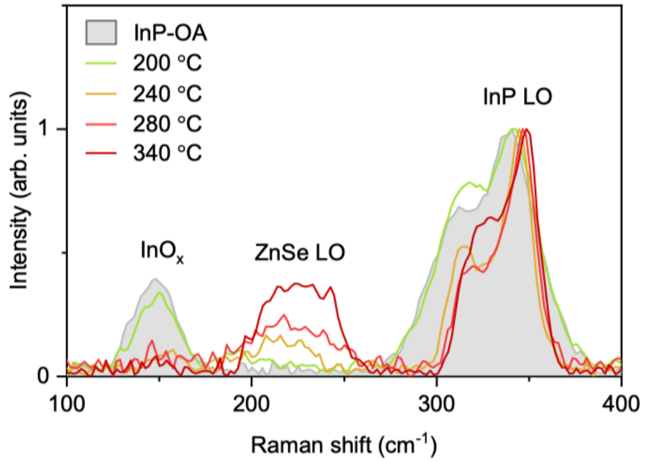

Supplementary Fig. 12| Raman spectroscopy of InP–ZnSe and InP/ZnSe based on HF-treated InP NCs. Pristine InP–OA (grey background), InP–ZnSe (yellow green), and InP/ZnSe based on HF-treated InP–OA at different annealing temperatures of 240 (orange), 280 (red), and 340 °C (deep red). The size of InP–OA used in these characterisations is 3.3 nm.

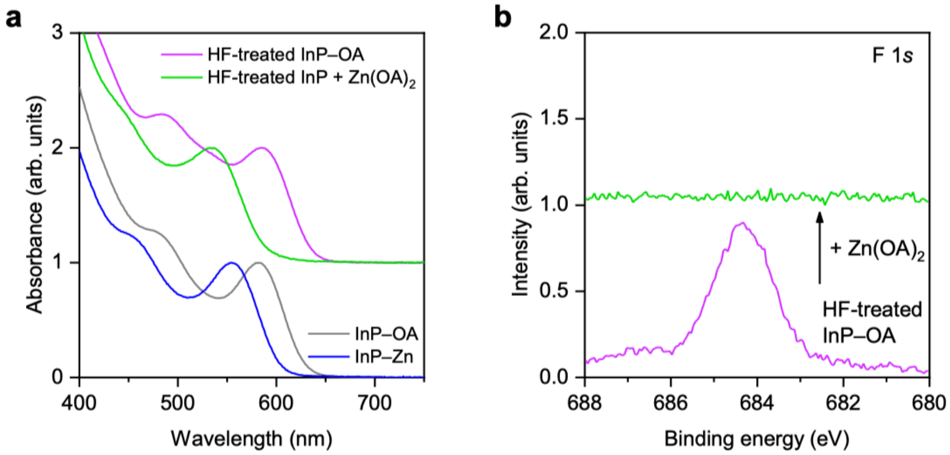

Supplementary Fig. 13| Removal of fluoride from HF-treated InP–OA using $Zn(OA)_2$. a, Absorption spectra of InP–OA (grey), InP–Zn (blue), and HF-treated InP–OA (magenta), and the addition of $Zn(OA)_2$ to HF-treated InP–OA at 200 °C for 10 min (green). **b**, F 1*s* electron binding energy for HF-treated InP–OA (magenta) and InP–Zn NCs based on HF-treated InP–OA (green). The size of the original InP–OA was 3.3 nm.

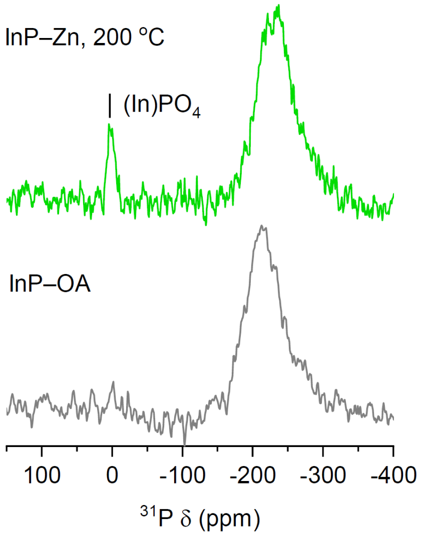

Supplementary Fig. 14| Increment of $(In)PO_4$ resonance after Zn-carboxylates treatment to InP–OA. Solid-state ^{31}P magic-angle spinning nuclear magnetic resonance spectra of pristine InP–OA (bottom) and InP–Zn (top). A clear P resonance signal of $(In)PO_4$ (at 2 ppm[7]) is observed after $Zn(OA)_2$ treatment.

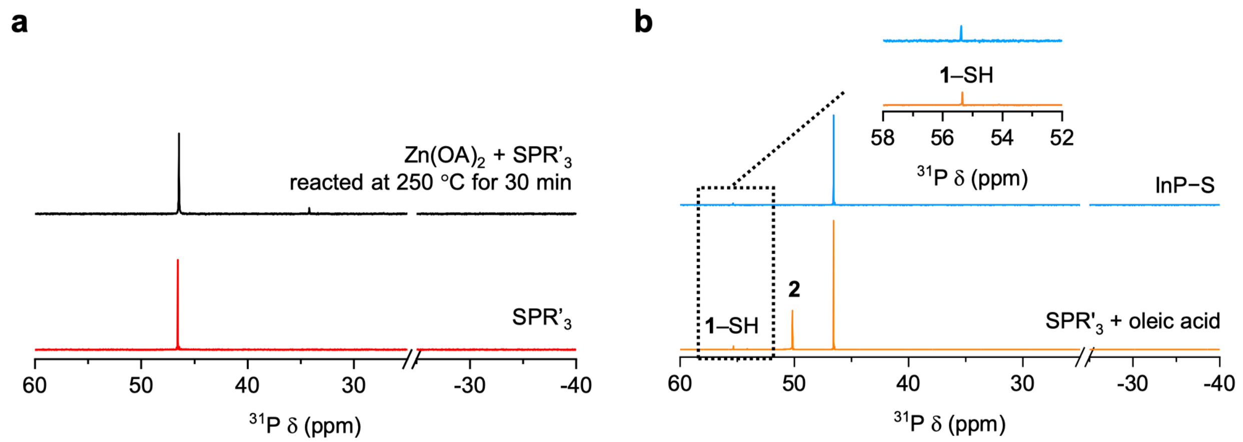

Supplementary Fig. 15| Stability of $Zn(OA)_2$ and SPR'$_3$ without InP NCs and Intermediate 1 formation on InP–OA by SPR'$_3$. a, ^{31}P NMR spectra of pristine SPR'$_3$ (bottom) and the mixture of $Zn(OA)_2$ and SPR'$_3$ reacted at 250 ºC for 30 min (top). The invariable SPR'$_3$ peak and absence of OPR'$_3$ imply that ZnS monomer formation is to be excluded under the given conditions. **b**, ^{31}P NMR spectra of the mixture of oleic acid and SPR'$_3$ reacted at 250 ºC for 30 min (bottom), and InP–S prepared under the same conditions (top). Inset magnifies the chemical shift corresponding to **1**–SH.

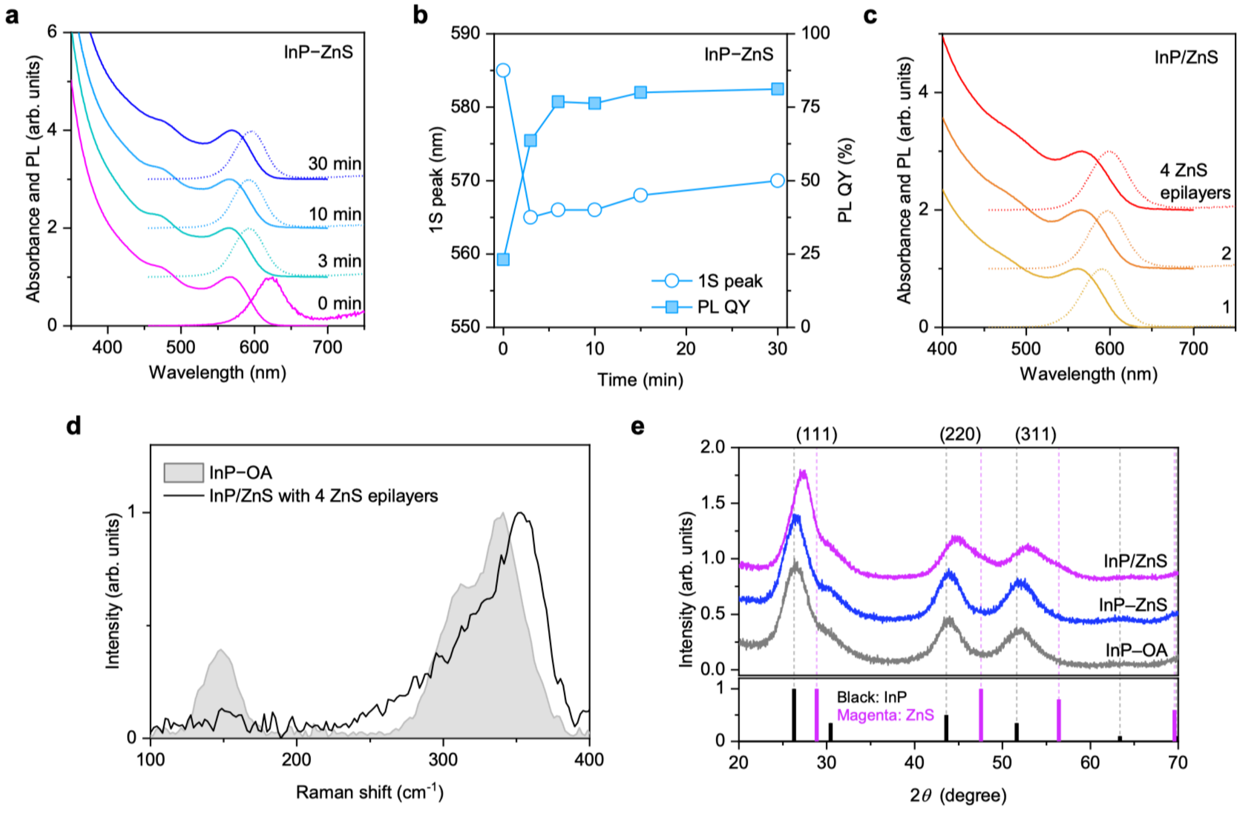

Supplementary Fig. 16| Characterisation of InP/ZnS NCs prepared through the surface-initiated heteroepitaxy process. a, Absorption (solid) and PL spectra (dotted), and **b**, 1S peak (open circle) and PLQY (closed square) of InP–ZnS at 250 °C as a function of the reaction time. HF-treated InP–OA was used. The 1S peak blueshift in the early stage is attributed to the In-to-Zn exchange, which reduces the effective size of the InP region. **c**, Absorption (solid) and PL spectra (dotted) of InP/ZnS NCs with different numbers of ZnS epilayers: 1 (yellow), 2 (orange), and 4 ZnS epilayers (red). **d**, Raman spectra of pristine InP-OA (grey background) and InP/ZnS NCs with 4 epitaxial layers (black). **e**, XRD diffractograms of InP–OA (grey), InP–ZnS (blue), and InP/ZnS with double epilayers (magenta). All experiments adopted InP–OA NCs with a size of 3.3 nm as starting materials.

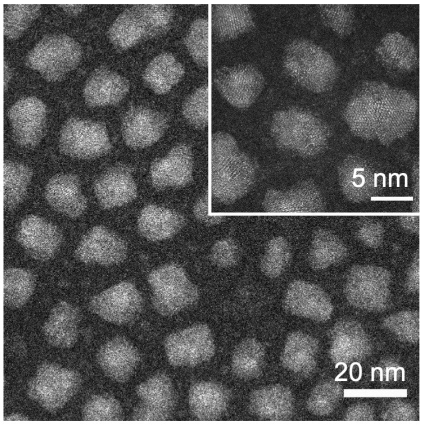

Supplementary Fig. 17| Dark-field transmission electron microscopy of InP/ZnS NCs with 2 ZnS epilayers fabricated by the proposed scheme. The inset shows a magnified image of InP/ZnS NCs with an average diameter of 4.7 ± 0.6 nm. The size of the original InP–OA NCs was 3.3 nm.

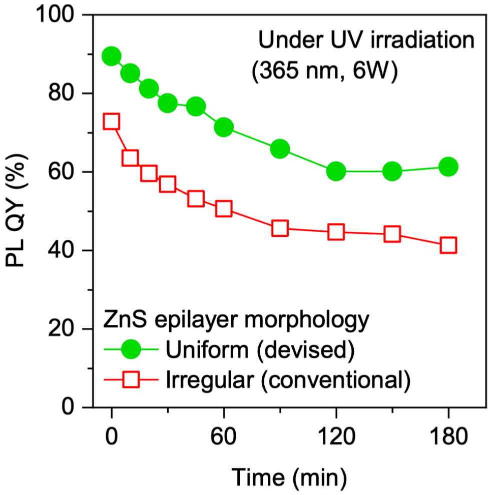

Supplementary Fig. 18| Photostability of InP/ZnS NCs. Temporal change in PLQY of two different InP/ZnS NCs with 4 ZnS epilayers under ultraviolet light irradiation (365 nm, 6 W) in air: uniform ZnS epilayer morphology using the proposed reaction scheme (green circle) and irregular ZnS morphology using the conventional high temperature scheme (red square). Optical density of the NCs dispersion was set to 0.5 at 450 nm.

Supplementary Table

Supplementary Table 1| Chemical composition of the NCs investigated in this study. The atomic ratios and numbers of In, P, and Se atoms in InP–OA, InP–Se, and InP–ZnSe, respectively, were determined using X-ray photoelectron spectroscopy. The detailed computations are described in Supplementary Note 1. The size of InP–OA was 2.4 nm.

	Atomic ratio / number of atoms		
	In	Se	P
InP–OA	1.44 / 215	-	1 / 149
InP–Se	1.45 / 216	0.50 / 74	1 / 149
InP–ZnSe at 0.5 min	1.40 / 209	0.30 / 45	1 / 149
InP–ZnSe at 30 min	1.09 / 163	1.14 / 170	1 / 149

Supplementary references

1 García-Rodríguez, R. & Liu, H. Mechanistic Study of the Synthesis of CdSe Nanocrystals: Release of Selenium. *Journal of the American Chemical Society* **134**, 1400-1403 (2012).

2 Liu, H., Owen, J. S. & Alivisatos, A. P. Mechanistic Study of Precursor Evolution in Colloidal Group II−VI Semiconductor Nanocrystal Synthesis. *Journal of the American Chemical Society* **129**, 305-312 (2007).

3 Förster, C., Veit, P., Ksenofontov, V. & Heinze, K. Diferrocenyl tosyl hydrazone with an ultrastrong NH···Fe hydrogen bond as double click switch. *Chemical Communications* **51**, 1514-1516 (2015).

4 García-Rodríguez, R., Hendricks, M. P., Cossairt, B. M., Liu, H. & Owen, J. S. Conversion Reactions of Cadmium Chalcogenide Nanocrystal Precursors. *Chemistry of Materials* **25**, 1233-1249 (2013).

5 Vahdati, M. & Tohidi Moghadam, T. Synthesis and Characterization of Selenium Nanoparticles-Lysozyme Nanohybrid System with Synergistic Antibacterial Properties. *Scientific Reports* **10**, 510 (2020).

6 Berengue, O. M. *et al.* Structural characterization of indium oxide nanostructures: a Raman analysis. *Journal of Physics D: Applied Physics* **43**, 045401 (2010).

7 Cros-Gagneux, A. *et al.* Surface Chemistry of InP Quantum Dots: A Comprehensive Study. *Journal of the American Chemical Society* **132**, 18147-18157 (2010).
